# A longitudinal resource for population neuroscience of school-age children and adolescents in China

**DOI:** 10.1038/s41597-023-02377-8

**Published:** 2023-08-21

**Authors:** Xue-Ru Fan, Yin-Shan Wang, Da Chang, Ning Yang, Meng-Jie Rong, Zhe Zhang, Ye He, Xiaohui Hou, Quan Zhou, Zhu-Qing Gong, Li-Zhi Cao, Hao-Ming Dong, Jing-Jing Nie, Li-Zhen Chen, Qing Zhang, Jia-Xin Zhang, Lei Zhang, Hui-Jie Li, Min Bao, Antao Chen, Jing Chen, Xu Chen, Jinfeng Ding, Xue Dong, Yi Du, Chen Feng, Tingyong Feng, Xiaolan Fu, Li-Kun Ge, Bao Hong, Xiaomeng Hu, Wenjun Huang, Chao Jiang, Li Li, Qi Li, Su Li, Xun Liu, Fan Mo, Jiang Qiu, Xue-Quan Su, Gao-Xia Wei, Yiyang Wu, Haishuo Xia, Chao-Gan Yan, Zhi-Xiong Yan, Xiaohong Yang, Wenfang Zhang, Ke Zhao, Liqi Zhu, for the Chinese Color Nest Consortium (CCNC), Ning Yang, Zhe Zhang, Ye He, Quan Zhou, Zhu-Qing Gong, Li-Zhi Cao, Hao-Ming Dong, Lei Zhang, Hui-Jie Li, Min Bao, Xu Chen, Yi Du, Xun Liu, Jiang Qiu, Xue-Quan Su, Gao-Xia Wei, Zhi-Xiong Yan, Xi-Nian Zuo, Xing-Ting Zhu, Xiao-Hui Hou, Yin-Shan Wang, Ping Wang, Yi-Wen Zhang, Dan-Yang Sui, Ting Xu, Zhi Yang, Lili Jiang, Ting-Yong Feng, Antao Chen, Ke Zhao, Yuan Zhou, Yan Zhuo, Zhentao Zuo, Li Ke, Fei Wang, F. Xavier Castellanos, Michael Peter Milham, Yu-Feng Zang, for the Lifespan Brain Chart Consortium (LBCC), Yin-Shan Wang, Ning Yang, Xi-Nian Zuo, Chris Adamson, Sophie Adler, Aaron F. Alexander-Bloch, Evdokia Anagnostou, Kevin M. Anderson, Ariosky Areces-Gonzalez, Duncan E. Astle, Bonnie Auyeung, Muhammad Ayub, Gareth Ball, Simon Baron-Cohen, Richard Beare, Saashi A. Bedford, Vivek Benegal, Richard A. I. Bethlehem, Frauke Beyer, Jong Bin Bae, John Blangero, Manuel Blesa Cábez, James P. Boardman, Matthew Borzage, Jorge F. Bosch-Bayard, Niall Bourke, Edward T. Bullmore, Vince D. Calhoun, Mallar M. Chakravarty, Christina Chen, Casey Chertavian, Gaël Chetelat, Yap S. Chong, Aiden Corvin, Manuela Costantino, Eric Courchesne, Fabrice Crivello, Vanessa L. Cropley, Jennifer Crosbie, Nicolas Crossley, Marion Delarue, Richard Delorme, Sylvane Desrivieres, Gabriel Devenyi, Maria A. Di Biase, Ray Dolan, Kirsten A. Donald, Gary Donohoe, Katharine Dunlop, Anthony D. Edwards, Jed T. Elison, Cameron T. Ellis, Jeremy A. Elman, Lisa Eyler, Damien A. Fair, Paul C. Fletcher, Peter Fonagy, Carol E. Franz, Lidice Galan-Garcia, Ali Gholipour, Jay Giedd, John H. Gilmore, David C. Glahn, Ian M. Goodyer, P. E. Grant, Nynke A. Groenewold, Faith M. Gunning, Raquel E. Gur, Ruben C. Gur, Christopher F. Hammill, Oskar Hansson, Trey Hedden, Andreas Heinz, Richard N. Henson, Katja Heuer, Jacqueline Hoare, Bharath Holla, Avram J. Holmes, Hao Huang, Kiho Im, Jonathan Ipser, Clifford R. Jack, Andrea P. Jackowski, Tianye Jia, David T. Jones, Peter B. Jones, Rene S. Kahn, Hasse Karlsson, Linnea Karlsson, Ryuta Kawashima, Elizabeth A. Kelley, Silke Kern, Ki-Woong Kim, Manfred G. Kitzbichler, William S. Kremen, François Lalonde, Brigitte Landeau, Jason Lerch, John D. Lewis, Jiao Li, Wei Liao, Deirel Paz-Linares, Conor Liston, Michael V. Lombardo, Jinglei Lv, Travis T. Mallard, Samuel R. Mathias, Machteld Marcelis, Bernard Mazoyer, Philip McGuire, Michael J. Meaney, Andrea Mechelli, Bratislav Misic, Sarah E. Morgan, David Mothersill, Cynthia Ortinau, Rik Ossenkoppele, Minhui Ouyang, Lena Palaniyappan, Leo Paly, Pedro M. Pan, Christos Pantelis, Min Tae M. Park, Tomas Paus, Zdenka Pausova, Alexa Pichet Binette, Karen Pierce, Xing Qian, Anqi Qiu, Armin Raznahan, Timothy Rittman, Amanda Rodrigue, Caitlin K. Rollins, Rafael Romero-Garcia, Lisa Ronan, Monica D. Rosenberg, David H. Rowitch, Giovanni A. Salum, Theodore D. Satterthwaite, H. Lina Schaare, Russell J. Schachar, Michael Schöll, Aaron P. Schultz, Jakob Seidlitz, David Sharp, Russell T. Shinohara, Ingmar Skoog, Christopher D. Smyser, Reisa A. Sperling, Dan J. Stein, Aleks Stolicyn, John Suckling, Gemma Sullivan, Benjamin Thyreau, Roberto Toro, Nicolas Traut, Kamen A. Tsvetanov, Nicholas B. Turk-Browne, Jetro J. Tuulari, Christophe Tzourio, Étienne Vachon-Presseau, Mitchell J. Valdes-Sosa, Pedro A. Valdes-Sosa, Sofie L. Valk, Therese van Amelsvoort, Simon N. Vandekar, Lana Vasung, Petra E. Vértes, Lindsay W. Victoria, Sylvia Villeneuve, Arno Villringer, Jacob W. Vogel, Konrad Wagstyl, Simon K. Warfield, Varun Warrier, Eric Westman, Margaret L. Westwater, Heather C. Whalley, Simon R. White, A. Veronica Witte, B. T. Thomas Yeo, Hyuk Jin Yun, Andrew Zalesky, Heather J. Zar, Anna Zettergren, Juan H. Zhou, Hisham Ziauddeen, Andre Zugman, Xi-Nian Zuo

**Affiliations:** 1 State Key Laboratory of Cognitive Neuroscience and Learning, Beijing Normal University, Beijing, 100875 China; 2 Department of Psychology, University of Chinese Academy of Sciences, Beijing, 100049 China; 3 Developmental Population Neuroscience Research Center, International Data Group/McGovern Institute for Brain Research, Beijing Normal University, Beijing, 100875 China; 4 CAS Key Laboratory of Behavioral Science, Institute of Psychology, Beijing, 100101 China; 5 College of Education, Hebei Normal University, Shijiazhuang, 050024 China; 6 School of Artificial Intelligence, Beijing University of Posts and Telecommunications, Beijing, 100876 China; 7 Laboratory of Cognitive Neuroscience and Education, School of Education Science, Nanning Normal University, Nanning, 530299 China; 8 Changping Laboratory, Beijing, 102206 China; 9 School of Government, Shanghai University of Political Science and Law, Shanghai, 201701 China; 10 School of Psychology, Research Center for Exercise and Brain Science, Shanghai University of Sport, Shanghai, 200438 China; 11 Faculty of Psychology, Southwest University, Chongqing, 400715 China; 12 NYU-ECNU Institute of Brain and Cognitive Science at New York University Shanghai, Shanghai, 200062 China; 13 Faculty of Arts and Science, New York University Shanghai, Shanghai, 200122 China; 14 State Key Laboratory of Brain and Cognitive Science, Institute of Psychology, Chinese Academy of Sciences, Beijing, 100101 China; 15 School of Psychology and Cognitive Science, East China Normal University, Shanghai, 200062 China; 16 Department of Psychology, Renmin University of China, Beijing, 100872 China; 17 Beijing Key Laboratory of Learning and Cognition, School of Psychology, Capital Normal University, Beijing, 100048 China; 18 School of Education, Hunan University of Science and Technology, Hunan Xiangtan, 411201 China; 19 National Basic Science Data Center, Beijing, 100190 China; 20 Flight Research Department, Aviation University of Air Force, Jilin Changchun, 130012 China; 21 Center for the Developing Brain, Child Mind Institute, New York, NY USA; 22 Affiliated Nanjing Brain Hospital, Nanjing Medical University, Nanjing, 210000 China; 23 Department of Child and Adolescent Psychiatry, New York University Grossman School of Medicine, New York, NY 10016 USA; 24 Nathan S. Kline Institute for Psychiatric Research, Orangeburg, New York, NY 10962 USA; 25 Institute of Psychological Sciences, Hangzhou Normal University, Hangzhou, 311121 China; 26 Developmental Imaging, Murdoch Children’s Research Institute, Melbourne, Victoria Australia; 27 Department of Medicine, Monash University, Melbourne, Victoria Australia; 28 UCL Great Ormond Street Institute for Child Health, 30 Guilford St, Holborn, London, WC1N 1EH UK; 29 Department of Psychiatry, University of Pennsylvania, Philadelphia, PA 19104 USA; 30 Department of Child and Adolescent Psychiatry and Behavioral Science, The Children’s Hospital of Philadelphia, Philadelphia, PA 19104 USA; 31 Lifespan Brain Institute, The Children’s Hospital of Philadelphia, Philadelphia, PA 19104 USA; 32 Department of Pediatrics University of Toronto, Toronto, Canada; 33 Holland Bloorview Kids Rehabilitation Hospital, Toronto, Canada; 34 Department of Psychology, Yale University, New Haven, CT USA; 35 The Clinical Hospital of Chengdu Brain Science Institute, MOE Key Lab for NeuroInformation, University of Electronic Science and Technology of China, No. 2006, Xiyuan Ave., West Hi-Tech Zone, Chengdu, 611731 China; 36 University of Pinar del Río “Hermanos Saiz Montes de Oca”, Pinar del Río, Cuba; 37 MRC Cognition and Brain Sciences Unit, University of Cambridge, Cambridge, UK; 38 Department of Psychology, School of Philosophy, Psychology and Language Sciences, University of Edinburgh, Edinburgh, United Kingdom; 39 Autism Research Centre, Department of Psychiatry, University of Cambridge, Cambridge, CB2 0SZ UK; 40 Queen’s University, Department of Psychiatry, Centre for Neuroscience Studies, Kingston, Ontario Canada; 41 University College London, Mental Health Neuroscience Research Department, Division of Psychiatry, London, UK; 42 Department of Paediatrics, University of Melbourne, Melbourne, Victoria Australia; 43 Cambridge Lifetime Asperger Syndrome Service (CLASS), Cambridgeshire and Peterborough NHS Foundation Trust, Cambridge, UK; 44 Centre for Addiction Medicine, National Institute of Mental Health and Neurosciences (NIMHANS), Bengaluru, 560029 India; 45 Brain Mapping Unit, Department of Psychiatry, University of Cambridge, Cambridge, CB2 0SZ UK; 46 Department of Neurology, Max Planck Institute for Human Cognitive and Brain Sciences, Leipzig, 04103 Germany; 47 Department of Neuropsychiatry, Seoul National University Bundang Hospital, Seongnam, Korea; 48 Department of Human Genetics, South Texas Diabetes and Obesity Institute, University of Texas Rio Grande Valley, Edinburg, USA; 49 MRC Centre for Reproductive Health, University of Edinburgh, Edinburg, UK; 50 Fetal and Neonatal Institute, Division of Neonatology, Children’s Hospital Los Angeles, Department of Pediatrics, Keck School of Medicine, University of Southern California, Los Angeles, California USA; 51 McGill Centre for Integrative Neuroscience, Ludmer Centre for Neuroinformatics and Mental Health, Montreal Neurological Institute, Montreal, Canada; 52 McGill University, Montreal, Canada; 53 Department of Brain Sciences, Imperial College London, London, UK; 54 Care Research & Technology Centre, UK Dementia Research Institute, London, UK; 55 Department of Psychiatry, University of Cambridge, Cambridge, CB2 0SZ UK; 56 Tri-institutional Center for Translational Research in Neuroimaging and Data Science, Georgia State University, Georgia Institute of Technology, and Emory University, Atlanta, GA USA; 57 Computational Brain Anatomy (CoBrA) Laboratory, Cerebral Imaging Centre, Douglas Mental Health University Institute, Verdun, Canada; 58 Penn Statistics in Imaging and Visualization Center, Department of Biostatistics, Epidemiology, and Informatics, Perelman School of Medicine, University of Pennsylvania, Philadelphia, PA USA; 59 Normandie Univ, UNICAEN, INSERM, U1237, PhIND “Physiopathology and Imaging of Neurological Disorders”, Institut Blood and Brain @ Caen-Normandie, Cyceron, 14000 Caen, France; 60 Singapore Institute for Clinical Sciences, Agency for Science, Technology and Research, Singapore, Singapore; 61 Department of Obstetrics and Gynaecology, Yong Loo Lin School of Medicine, National University of Singapore, Singapore, Singapore; 62 Department of Psychiatry, Trinity College, Dublin, Ireland; 63 Cerebral Imaging Centre, Douglas Mental Health University Institute, Verdun, Canada; 64 Undergraduate program in Neuroscience, McGill University, Montreal, Canada; 65 Department of Neuroscience, University of California, San Diego, San Diego, CA 92093 USA; 66 Autism Center of Excellence, University of California, San Diego, San Diego, CA 92037 USA; 67 Institute of Neurodegenerative Disorders, CNRS UMR5293, CEA, University of Bordeaux, Bordeaux, France; 68 Melbourne Neuropsychiatry Centre, University of Melbourne, Melbourne, Australia; 69 The Hospital for Sick Children, Toronto, Canada; 70 Department of Psychiatry, School of Medicine, Pontificia Universidad Católica de Chile, Diagonal Paraguay 362, Santiago, 8330077 Chile; 71 Department of Psychiatry, University of Oxford, OX3 7JX Oxford, UK; 72 Child and Adolescent Psychiatry Department, Robert Debré University Hospital, AP-HP, F-75019 Paris, France; 73 Human Genetics and Cognitive Functions, Institut Pasteur, F-75015 Paris, France; 74 Social, Genetic and Developmental Psychiatry Centre, Institute of Psychiatry, Psychology & Neuroscience, King’s College London, London, UK; 75 Cerebral Imaging Centre, Douglas Mental Health University Institute, Montreal, QC, Canada, McGill Department of Psychiatry, Montreal, QC Canada; 76 Department of Psychiatry, McGill University, Montreal, QC Canada; 77 Department of Psychiatry, Brigham and Women’s Hospital, Harvard Medical School, Boston, Massachusetts USA; 78 Max Planck UCL Centre for Computational Psychiatry and Ageing Research, University College London, London, UK; 79 Wellcome Centre for Human Neuroimaging, University College London, London, UK; 80 Wellcome Centre for Human Neuroimaging, 12 Queen Square, London, WC1N 3AR UK; 81 Division of Developmental Paediatrics, Department of Paediatrics and Child Health, Red Cross War Memorial Children’s Hospital, Klipfontein Road/Private Bag, Rondebosch, 7700/7701 Cape Town, South Africa; 82 Neuroscience Institute, University of Cape Town, Cape Town, South Africa; 83 Center for Neuroimaging, Cognition & Genomics (NICOG), School of Psychology, National University of Ireland Galway, Galway, Ireland; 84 Weil Family Brain and Mind Research Institute, Department of Psychiatry, Weill Cornell Medicine, New York, USA; 85 Centre for the Developing Brain, King’s College London, London, UK; 86 Evelina London Children’s Hospital, London, UK; 87 MRC Centre for Neurodevelopmental Disorders, London, UK; 88 Institute of Child Development, Department of Pediatrics, Masonic Institute for the Developing Brain, University of Minnesota, Minneapolis, MN USA; 89 Haskins Laboratories, New Haven, CT USA; 90 Department of Psychiatry, Center for Behavior Genetics of Aging, University of California, San Diego, La Jolla, CA USA; 91 Desert-Pacific Mental Illness Research Education and Clinical Center, VA San Diego Healthcare, San Diego, CA USA; 92 Department of Psychiatry, University of California San Diego, Los Angeles, CA USA; 93 Department of Psychiatry, University of Cambridge, and Wellcome Trust MRC Institute of Metabolic Science, Cambridge Biomedical Campus, Cambridge, UK; 94 Cambridgeshire and Peterborough NHS Foundation Trust, Cambridge, USA; 95 Department of Clinical, Educational and Health Psychology, University College London, London, UK; 96 Anna Freud National Centre for Children and Families, London, UK; 97 Department of Psychiatry, Center for Behavior Genetics of Aging, University of California, San Diego, La Jolla, CA 92093 USA; 98 Cuban Center for Neuroscience, La Habana, Cuba; 99 Computational Radiology Laboratory, Boston Children’s Hospital, Boston, MA 02115 USA; 100 Department of Child and Adolescent Psychiatry, University of California, San Diego, San Diego, CA 92093 USA; 101 Department of Psychiatry, University of California San Diego, San Diego, CA USA; 102 Department of Psychiatry, University of North Carolina, Chapel Hill, NC USA; 103 Department of Psychiatry, Boston Children’s Hospital and Harvard Medical School, Boston, MA 02115 USA; 104 Harvard Medical School, Boston, MA 02115 USA; 105 Division of Newborn Medicine and Neuroradiology, Fetal Neonatal Neuroimaging and Developmental Science Center, Boston Children’s Hospital, Harvard Medical School, Boston, MA 02115 USA; 106 Department of Paediatrics and Child Health, Red Cross War Memorial Children’s Hospital, SA-MRC Unit on Child & Adolescent Health, University of Cape Town, Cape Town, South Africa; 107 Weill Cornell Institute of Geriatric Psychiatry, Department of Psychiatry, Weill Cornell Medicine, New York, USA; 108 Lifespan Brain Institute, The Children’s Hospital of Philadelphia, Philadelphia, PA 19105 USA; 109 Mouse Imaging Centre, Toronto, Canada; 110 Clinical Memory Research Unit, Department of Clinical Sciences Malmö, Lund University, Malmö, Sweden; 111 Memory Clinic, Skåne University Hospital, Malmö, Sweden; 112 Department of Neurology, Icahn School of Medicine at Mount Sinai, New York, NY 10029 USA; 113 Athinoula A. Martinos Center for Biomedical Imaging, Department of Radiology, Massachusetts General Hospital, Harvard Medical School, Boston, MA 02129 USA; 114 Department of Psychiatry and Psychotherapy, Charite University Hospital Berlin, Berlin, Germany; 115 Department of Psychiatry, University of Cambridge, Cambridge, UK; 116 Institut Pasteur, Université Paris Cité, Unité de Neuroanatomie Appliquée et Théorique, F-75015 Paris, France; 117 Department of Psychiatry, University of Cape Town, Cape Town, South Africa; 118 Department of Integrative Medicine, NIMHANS, Bengaluru, 560029 India; 119 Accelerator Program for Discovery in Brain disorders using Stem cells (ADBS), Department of Psychiatry, NIMHANS, Bengaluru, 560029 India; 120 Department of Psychiatry, Brain Health Institute, Rutgers University, Piscataway, NJ USA; 121 Department of Radiology, Children’s Hospital of Philadelphia and University of Pennsylvania, Philadelphia, PA 19104 USA; 122 Division of Newborn Medicine, Fetal Neonatal Neuroimaging and Developmental Science Center, Boston Children’s Hospital, Harvard Medical School, Boston, MA 02115 USA; 123 Boston Children’s Hospital, Boston, MA 02115 USA; 124 Department of Psychiatry and Mental Health, Clinical Neuroscience Institute, University of Cape Town, Cape Town, South Africa; 125 Department of Radiology, Mayo Clinic, Rochester, MN 55905 USA; 126 Department of Psychiatry, Universidade Federal de São Paulo, São Paulo, Brazil; 127 National Institute of Developmental Psychiatry, CNPq, São Paulo, Brazil; 128 Institute of Science and Technology for Brain-Inspired Intelligence, Fudan University, Shanghai, 200433 China; 129 Key Laboratory of Computational Neuroscience and BrainInspired Intelligence (Fudan University), Ministry of Education, Shanghai, China; 130 Centre for Population Neuroscience and Precision Medicine (PONS), Institute of Psychiatry, Psychology and Neuroscience, SGDP Centre, King’s College London, London, SE5 8AF UK; 131 Department of Neurology, Mayo Clinic, Rochester, MN USA; 132 Department of Radiology, Mayo Clinic, Rochester, MN USA; 133 Cambridgeshire and Peterborough NHS Foundation Trust, Huntingdon, United Kingdom; 134 Department of Psychiatry, Icahn School of Medicine at Mount Sinai, New York, NY USA; 135 Department of Psychiatry, Icahn School of Medicine, Mount Sinai, New York, USA; 136 Department of Clinical Medicine, Department of Psychiatry and Turku Brain and Mind Center, FinnBrain Birth Cohort Study, University of Turku and Turku University Hospital, Turku, Finland; 137 Centre for Population Health Research, Turku University Hospital and University of Turku, Turku, Finland; 138 Institute of Development, Aging and Cancer, Tohoku University, Seiryocho, Aobaku, Sendai, 980-8575 Japan; 139 Queen’s University, Departments of Psychology and Psychiatry, Centre for Neuroscience Studies, Kingston, Ontario Canada; 140 Neuropsychiatric Epidemiology Unit, Department of Psychiatry and Neurochemistry, Institute of Neuroscience and Physiology, the Sahlgrenska Academy, Centre for Ageing and Health (AGECAP) at the University of Gothenburg, Gothenburg, Sweden; 141 Region Vöstra Götaland, Sahlgrenska University Hospital, Psychiatry, Cognition and Old Age Psychiatry Clinic, Gothenburg, Sweden; 142 Department of Brain and Cognitive Sciences, Seoul National University College of Natural Sciences, Seoul, Republic of Korea; 143 Department of Neuropsychiatry, Seoul National University Bundang Hospital, Seongnam, Republic of Korea; 144 Department of Psychiatry, Seoul National University College of Medicine, Seoul, Republic of Korea; 145 Department of Brain and Cognitive Science, Seoul National University College of Natural Sciences, Seoul, South Korea; 146 Section on Developmental Neurogenomics, Human Genetics Branch, National Institute of Mental Health, Bethesda, MD USA; 147 Department of Medical Biophysics, University of Toronto, Toronto, ON Canada; 148 Mouse Imaging Centre, The Hospital for Sick Children, Toronto, ON Canada; 149 Wellcome Centre for Integrative Neuroimaging, FMRIB, Nuffield Department of Clinical Neuroscience, University of Oxford, Oxford, UK; 150 Montreal Neurological Institute, McGill University, Montreal, Canada; 151 The Clinical Hospital of Chengdu Brain Science Institute, University of Electronic Science and Technology of China, Chengdu, 611731 China; 152 Cuban Neuroscience Center, Havana, Cuba; 153 Department of Psychiatry and Brain and Mind Research Institute, Weill Cornell Medicine, New York, USA; 154 Laboratory for Autism and Neurodevelopmental Disorders, Center for Neuroscience and Cognitive Systems @UniTn, Istituto Italiano di Tecnologia, Rovereto, Italy; 155 School of Biomedical Engineering & Brain and Mind Centre, The University of Sydney, Sydney, NSW Australia; 156 Department of Psychology, University of Texas, Austin, Texas 78712 USA; 157 Department of Psychiatry and Neuropsychology, School of Mental Health and Neuroscience, EURON, Maastricht University Medical Centre, PO Box 616, 6200 MD Maastricht, the Netherlands; 158 Institute for Mental Health Care Eindhoven (GGzE), Eindhoven, the Netherlands; 159 Bordeaux University Hospital, Bordeaux, France; 160 Department of Psychosis Studies, Institute of Psychiatry, Psychology and Neuroscience, King’s College London, London, UK; 161 Ludmer Centre for Neuroinformatics and Mental Health, Douglas Mental Health University Institute, McGill University, Montreal, Quebec Canada; 162 Singapore Institute for Clinical Sciences, Singapore, Singapore; 163 McConnell Brain Imaging Centre, Montreal Neurological Institute, McGill University, Montreal, QC H3A 2B4 Canada; 164 Department of Computer Science and Technology, University of Cambridge, Cambridge, CB3 0FD UK; 165 The Alan Turing Institute, London, NW1 2DB UK; 166 Department of Psychology, School of Business, National College of Ireland, Dublin, Ireland; 167 School of Psychology & Center for Neuroimaging and Cognitive Genomics, National University of Ireland Galway, Galway, Ireland; 168 Department of Psychiatry, Trinity College Dublin, Dublin, Ireland; 169 Department of Pediatrics, Washington University in St. Louis, St. Louis, Missouri USA; 170 Alzheimer Center Amsterdam, Department of Neurology, Amsterdam Neuroscience, Vrije Universiteit Amsterdam, Amsterdam UMC, Amsterdam, The Netherlands; 171 Lund University, Clinical Memory Research Unit, Lund, Sweden; 172 Robarts Research Institute & The Brain and Mind Institute, University of Western Ontario, London, Ontario Canada; 173 Department of Psychiatry, Federal University of Sao Poalo (UNIFESP), São Paulo, Brazil; 174 National Institute of Developmental Psychiatry for Children and Adolescents (INPD), São Paulo, Brazil; 175 Melbourne Neuropsychiatry Centre, Department of Psychiatry, The University of Melbourne and Melbourne Health, Carlton South, Victoria Australia; 176 Melbourne School of Engineering, The University of Melbourne, Parkville, Victoria Australia; 177 Florey Institute of Neuroscience and Mental Health, Parkville, VIC Australia; 178 Department of Psychiatry, Schulich School of Medicine and Dentistry, Western University, London, ON Canada; 179 Department of Psychiatry, Faculty of Medicine and Centre Hospitalier Universitaire Sainte-Justine, University of Montreal, Montreal, Quebec Canada; 180 Departments of Psychiatry and Psychology, University of Toronto, Toronto, ON Canada; 181 Departments of Physiology and Nutritional Sciences, University of Toronto, Toronto, Canada; 182 Department of Psychiatry, Faculty of Medicine, McGill University, Montreal, Qc H3A 1Y2 Canada; 183 Douglas Mental Health University Institute, Montreal, Qc H4H 1R3 Canada; 184 Department of Neurosciences, University of California, San Diego La Jolla, CA USA; 185 Center for Sleep and Cognition, Yong Loo Lin School of Medicine, National University of Singapore, Singapore, Singapore; 186 Department of Biomedical Engineering, The N.1 Institute for Health, National University of Singapore, Singapore, Singapore; 187 Department of Clinical Neurosciences, University of Cambridge, Cambridge, UK; 188 Department of Neurology, Harvard Medical School, Boston, USA; 189 Department of Neurology, Boston Children’s Hospital, Boston, MA 02115 USA; 190 Instituto de Biomedicina de Sevilla (IBiS) HUVR/CSIC/Universidad de Sevilla, Dpto. de Fisiología Médica y Biofésica, Sevilla, Spain; 191 Department of Psychology, Neuroscience Institute, University of Chicago, Chicago, USA; 192 Department of Paediatrics and Wellcome-MRC Cambridge Stem Cell Institute, University of Cambridge, Hills Road, Cambridge, UK; 193 Department of Psychiatry, Universidade Federal do Rio Grande do Sul (UFRGS), Porto Alegre, Brazil; 194 National Institute of Developmental Psychiatry (INPD), São Paulo, Brazil; 195 Lifespan Informatics & Neuroimaging Center, University of Pennsylvania, Philadelphia, PA 19104 USA; 196 Otto Hahn Group Cognitive Neurogenetics, Max Planck Institute for Human Cognitive and Brain Sciences, Leipzig, Germany; 197 Institute of Neuroscience and Medicine (INM-7: Brain and Behaviour), Research Centre Juelich, Juelich, Germany; 198 Wallenberg Centre for Molecular and Translational Medicine, University of Gothenburg, Gothenburg, Sweden; 199 Department of Psychiatry and Neurochemistry, University of Gothenburg, Gothenburg, Sweden; 200 Dementia Research Centre, Queen’s Square Institute of Neurology, University College London, London, UK; 201 Harvard Aging Brain Study, Department of Neurology, Massachusetts General Hospital, Boston, MA 02114 USA; 202 Athinoula A. Martinos Center for Biomedical Imaging, Department of Radiology, Massachusetts General Hospital, Charlestown, MA 02129 USA; 203 Care Research & Technology Centre, UK Dementia Research Institute, London, UK; 204 Center for Biomedical Image Computing and Analytics, Department of Radiology, Perelman School of Medicine, University of Pennsylvania, Philadelphia, PA USA; 205 Departments of Neurology, Pediatrics, and Radiology, Washington University School of Medicine, St. Louis, USA; 206 Center for Alzheimer Research and Treatment, Department of Neurology, Brigham and Women’s Hospital, Boston, MA 02115 USA; 207 SA MRC Unit on Risk & Resilience in Mental Disorders, Dept of Psychiatry and Neuroscience Institute, University of Cape Town, Cape Town, South Africa; 208 Division of Psychiatry, Centre for Clinical Brain Sciences, University of Edinburgh, Edinburgh, UK; 209 Cambridge and Peterborough Foundation NHS Trust, Peterborough, UK; 210 Université de Paris, Paris, France; 211 Department of Neuroscience, Institut Pasteur, Paris, France; 212 Center for Research and Interdisciplinarity (CRI), Université Paris Descartes, Paris, France; 213 Department of Psychology, University of Cambridge, Cambridge, UK; 214 Wu Tsai Institute, Yale University, New Haven, CT USA; 215 Department of Clinical Medicine, Department of Psychiatry, FinnBrain Birth Cohort Study, University of Turku, Turku, Finland; 216 Department of Clinical Medicine, University of Turku, Turku, Finland; 217 Turku Collegium for Science, Medicine and Technology, University of Turku, Turku, Finland; 218 Univ. Bordeaux, Inserm, Bordeaux Population Health Research Center, U1219, CHU Bordeaux, F-33000 Bordeaux, France; 219 Faculty of Dental Medicine and Oral Health Sciences, McGill University, Montreal, Qc H3A 1G1 Canada; 220 Faculty of Dentistry, McGill University, Montreal, Qc H3A 1G1 Canada; 221 Alan Edwards Centre for Research on Pain (AECRP), McGill University, Montreal, Qc H3A 1G1 Canada; 222 Joint China-Cuba Lab,University of Electronic Science and Technology, Chengdu China/Cuban Center for Neuroscience, La Habana, Cuba; 223 University of Electronic Science and Technology of China/Cuban Center for Neuroscience, Chengdu, China; 224 Institute for Neuroscience and Medicine 7, Forschungszentrum Juelich; Max Planck Institute for Human Cognitive and Brain Sciences, Leipzig, Germany; 225 Department of Psychiatry & Neurosychology, Maastricht University, Maastricht, The Netherlands; 226 Department of Biostatistics, Vanderbilt University, Nashville, Tennessee USA; 227 Department of Biostatistics, Vanderbilt University Medical Center, Nashville, Tennessee USA; 228 Division of Newborn Medicine, Fetal Neonatal Neuroimaging and Developmental Science Center, Department of Pediatrics, Boston Children’s Hospital, Boston, MA 02115 USA; 229 McConnell Brain Imaging Center, Montreal Neurological Institute, McGill University, Montreal, Quebec Canada; 230 Clinic for Cognitive Neurology, University of Leipzig Medical Center, Leipzig, 04103 Germany; 231 Wellcome Centre for Human Neuroimaging, Institute of Neurology, University College London, WC1N 3AR London, UK; 232 Division of Clinical Geriatrics, Center for Alzheimer Research, Department of Neurobiology, Care Sciences and Society, Karolinska Institutet, Stockholm, Sweden; 233 Generation Scotland, University of Edinburgh, Edinburgh, UK; 234 MRC Biostatistics Unit, University of Cambridge, Cambridge, England; 235 Faculty of Medicine, CRC 1052 ‘Obesity Mechanisms’, University of Leipzig, Leipzig, 04103 Germany; 236 Department of Electrical and Computer Engineering, National University of Singapore, Singapore, Singapore; 237 Centre for Sleep & Cognition and Centre for Translational MR Research, Yong Loo Lin School of Medicine, National University of Singapore, Singapore, Singapore; 238 N.1 Institute for Health & Institute for Digital Medicine, National University of Singapore, Singapore, Singapore; 239 Integrative Sciences and Engineering Programme (ISEP), National University of Singapore, Singapore, Singapore; 240 Fetal Neonatal Neuroimaging and Developmental Science Center, Division of Newborn Medicine, Boston Children’s Hospital, Harvard Medical School, Boston, MA 02115 USA; 241 Melbourne Neuropsychiatry Centre, University of Melbourne, Melbourne, Australia; Department of Biomedical Engineering, University of Melbourne, Melbourne, Australia; 242 SAMRC Unit on Child & Adolescent Health, University of Cape Town, Cape Town, South Africa; 243 Center for Translational Magnetic Resonance Research, Yong Loo Lin School of Medicine, National University of Singapore, Singapore, Singapore; 244 Wellcome Trust-MRC Institute of Metabolic Science, University of Cambridge, Cambridge, CB2 0SZ UK; 245 Cambridgeshire and Peterborough Foundation Trust, Cambridge, CB21 5EF UK; 246 National Institute of Mental Health (NIMH), National Institutes of Health (NIH), Bethesda, Maryland USA; 247 Department of Psychiatry, Escola Paulista de Medicina, São Paulo, Brazil

**Keywords:** Cognitive neuroscience

## Abstract

During the past decade, cognitive neuroscience has been calling for population diversity to address the challenge of validity and generalizability, ushering in a new era of population neuroscience. The developing Chinese Color Nest Project (devCCNP, 2013–2022), the first ten-year stage of the lifespan CCNP (2013–2032), is a two-stages project focusing on brain-mind development. The project aims to create and share a large-scale, longitudinal and multimodal dataset of typically developing children and adolescents (ages 6.0–17.9 at enrolment) in the Chinese population. The devCCNP houses not only phenotypes measured by demographic, biophysical, psychological and behavioural, cognitive, affective, and ocular-tracking assessments but also neurotypes measured with magnetic resonance imaging (MRI) of brain morphometry, resting-state function, naturalistic viewing function and diffusion structure. This Data Descriptor introduces the first data release of devCCNP including a total of 864 visits from 479 participants. Herein, we provided details of the experimental design, sampling strategies, and technical validation of the devCCNP resource. We demonstrate and discuss the potential of a multicohort longitudinal design to depict normative brain growth curves from the perspective of developmental population neuroscience. The devCCNP resource is shared as part of the “Chinese Data-sharing Warehouse for *In-vivo* Imaging Brain” in the *Chinese Color Nest Project (CCNP) – Lifespan Brain-Mind Development Data Community* (https://ccnp.scidb.cn) at the Science Data Bank.

## Background & Summary

To explore the relationship between human behaviour and the brain, especially with respect to individual differences and precision medicine, large-scale neuroimaging data collection is necessary. In 2008, thirty-five laboratories from 10 countries including China, launched the 1000 Functional Connectomes Project (FCP)^1^. This global project shared MRI data from 1,414 worldwide participants’ neuroimaging data through the Network Information Technology Resources Collaboratory (NITRC) in the United States. As a milestone in open science for human brain function, the project demonstrated the association of individual differences in functional connectivity with demographic phenotypes (age and sex)^1^. Since then, population-based prospective efforts have been implemented by worldwide brain initiatives, such as Human Connectome Project (HCP)^2^, US BRAIN Initiative (Brain Research through Advancing Innovative Neurotechnologies Initiative, or BRAIN)^3^, Brain Mapping by Integrated Neurotechnologies for Disease Studies (Brain/MINDS) in Japan^4^, UK (United Kingdom) Biobank^5^, BRAIN Canada^6^, and Adolescent Brain Cognitive Development (ABCD) study^7^. This has introduced big data into cognitive neuroscience with population imaging, namely population neuroscience^8,9^, to increase population diversity or sample representativeness for improvements in generalizability, a significant challenge faced by current cognitive neuroscience research^10,11^.

The Chinese Color Nest Project (CCNP, 2013–2032)^12^ is an early representative effort, likely the first in China, investigating brain growth during the transition period from childhood to adolescence. CCNP has built and accumulated rich and valuable experiences as a pilot study to accelerate the pace of initiating related brain-mind development cohort studies in the China Brain Project^13,14^. CCNP is devoted to collecting nationwide data on brain structure and function across different stages of human lifespan development (6–85 years old). The long-term goal of this work is to create neurobiologically sound developmental curves for the brain to characterize phenomenological changes associated with the onset of various forms of mental health and learning disorders, as well as to predict the developmental status (i.e., age-expected values) of an individual brain’s structure or function. The developmental component of CCNP (devCCNP), also known as “Growing Up in China”^15^, has established follow-up cohorts in Chongqing and Beijing, China. With the collection of longitudinal brain images and psychobehavioural samples from school-age children and adolescents (6–18 years) in multiple cohorts, devCCNP has constructed a full set of school-age brain templates, morphological growth curves^16^ and functional connectivity gradients^17^ for the Chinese Han population as well as related (although preliminary) differences in brain development between Chinese and American school-age children^16^. The project has contributed to charting human brain development across the lifespan (0–100 years) in an international teamwork led by the Lifespan Brain Chart Consortium (LBCC)^18^.

To expand available resources for investigating population diversity^19^ while recognizing and addressing the issues of sampling bias, and inclusion barriers within developmental population neuroscience^20^, we describe and share the brain-mind datasets of devCCNP here. We offer a comprehensive outline of the devCCNP protocol, along with recommendations to ensure that devCCNP can be scaled up to facilitate access to more diverse populations in the future. We provide all the anonymized raw data adhering to Brain Imaging Data Structure (BIDS) standards^21^. In summary, this dataset comprises ample tasks addressing neurodevelopmental milestones of both primary and higher-order cognitive functions. The dataset holds the potential to deepen our understanding of brain development in various dimensions, and augments assessments of cultural diversity among the existing datasets using accelerated longitudinal designs (ALD) (see Table 1 from the cohort profile on CCNP^12^ for a nonexhaustive list of normative developmental samples obtained by ALD). In addition, we hope that the devCCNP will provide a resource to explore potential regional differences due to multisite sampling, and their impacts on brain development.

**Table 1:** Examples of two individual schedule for each wave’s data collection.

Example of 2-visit Schedule		
	Day 1	Day 2
09 AM	Demographics & Characteristics	Intelligence Quotient Measure
	Mock MRI Scan	
10 AM	Biophysical Measures	
	Magnetic Resonance Imaging	
11 AM		Psychological Behaviour Tasks/Tests Part2
	Lunch Break	
12 AM		
13 AM		
	Psychological Behaviour Questionnaires	Psychological Behaviour Tasks/Tests Part4
14 AM		
	Psychological Behaviour Tasks/Tests Part1	
15 AM		
16 AM		Physical Fitness Measures
**Example of 4-visit Schedule**		
	**Day 1**	**Day 2**
09 AM	Demographics & Characteristics	Psychological Behaviour Questionnaires
	Mock MRI Scan	
10 AM	Biophysical Measures	Psychological Behaviour Tasks/Tests Part1
	Magnetic Resonance Imaging	
11 AM		
	**Day 3**	**Day 4**
09 AM	Psychological Behaviour Tasks/Tests Part2	Intelligence Quotient Measure
10 AM		
11 AM		Physical Fitness Measures

## Methods

### Overall design

**Fig. 1:**
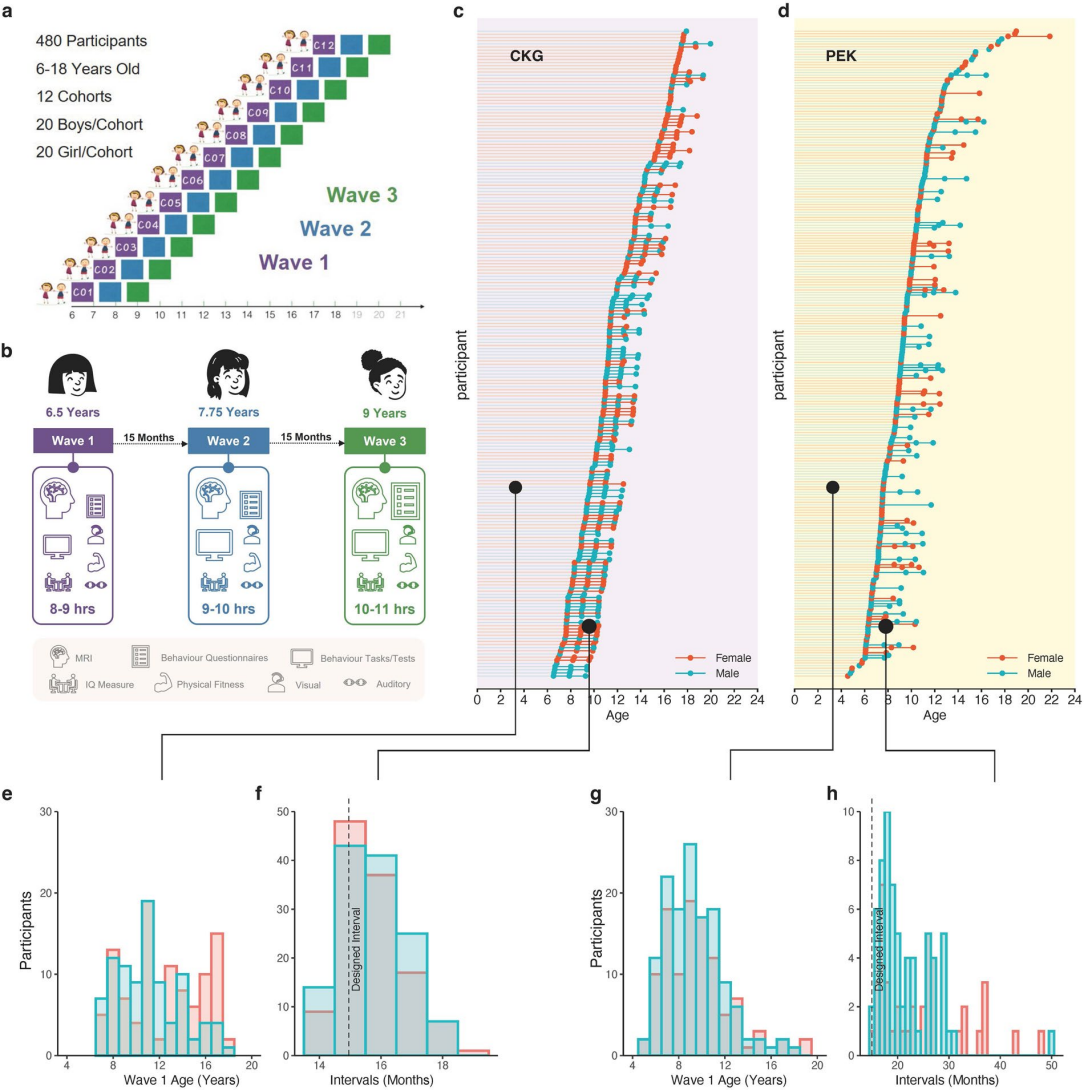
Experimental design and sample composition. (**a**) The Accelerated Longitudinal Design (ALD) of devCCNP has 3 repeated measuring waves: Wave 1 (baseline, purple), Wave 2 (follow-up 1, blue), and Wave 3 (follow-up 2, green). The age range of participant enrolment was 6–18 years. The 480 participants were divided into 12 age cohorts, with 20 boys and 20 girls in each. The interval between each successive waves was designed to be 15 months. (**b**) An example of a participant’s protocol who enrolled at 6.5 years. Measurement content is justified according to the age of each participant. As shown in Supplementary Table S1, the number of psychological behaviour measurements (related to questionnaires and computer-mediated tasks/tests) increases with age. (**c,d**) Age and sex distributions for participants’ completion in the CKG and PEK Samples (female, red; male, blue). Dots indicate the specific age of each wave’s data collection, while lines indicate the actual intervals between two waves. (**e,g**) Numbers of participants enrolled (Wave 1) in each age group are calculated according to sex. (**f**) The actual intervals in the CKG Sample better adhere to the original design; the largest interval is 19 months. (**h**) In the PEK Sample, intervals have been commonly extended from 16 to 50 months.

The first stage of devCCNP aimed to establish an ALD cohort. The cohort consisted of 480 participants with typical development, who were evenly divided into age-specific groups. Each age cohort contain 20 boys and 20 girls (Fig. 1a). We conducted data collection in two regions in China with distinct geographic and socioeconomic profiles, the Beibei District of Chongqing (devCCNP-CKG Sample) and the Chaoyang District of Beijing(devCCNP-PEK Sample), to capture a more representative sample of the Chinese population and its diverse characteristics. The devCCNP-CKG Sample was collected from March 2013 to January 2017, and the devCCNP-PEK Sample was collected from September 2017 to December 2022. Participants underwent assessment three times in total, referred to as three “waves” of visits. To account for season effects, there was a 15-month time gap between each wave (Fig. 1b). A repeated protocol was applied, which was adjusted based on the participants’ age. The total time for each assessment was approximately 10–12 hours, including preparation time and short breaks. The time duration of each visit is listed in Supplementary Table S1.

### Recruitment strategy

The devCCNP project focused on enrolling typically developing school-age Chinese children and adolescents. The CKG Sample was included one primary school and one junior high school in Chongqing. The participants were recruited through face-to-face communications between parents, schools, and CCNP program staff. In the case of the PEK Sample, recruitment took place in Beijing, where community-based recruitment was initially accomplished through various science popularization activities and online advertisements. We provided a series of activities for the families to experience educational neuroscience, including lectures on the brain, neuroimaging, cognitive neuroscience, and facility tours to experience MRI mock scanning, to make them interested and familiar with the entire procedure. Because the project gradually gained a good reputation, word of mouth recruitment became a major source of participants.

### Retention strategy

To accommodate each participant’s after-school schedule, the experimental procedures for one wave were conducted in 2 to 4 separate visits as shown in Table 1. A 1-month time window was given for completing all the experimental protocols in one wave, allowing for flexibility in scheduling. During the COVID-19 pandemic, relevant to the PEK Sample only, the time window was extended to three months to ensure that participants were able to complete the study. In addition, we offered modest monetary compensation and a variety of educational toys to the participants. The primary strategies to promote retention are listed below.

#### Personal development report

After each wave’s data collection, every participant was provided with a well-designed personal development report containing feedback on various aspects of physiological characteristics (e.g. height, weight, blood pressure, and heart rate), cognitive ability (e.g. intelligence quotient or IQ), social-emotional development (e.g. social anxiety, depression, stress perception and behavioural problems), personality, and brain development. The brain development report included measurements of global and network morphology (i.e., 7 large-scale brain network organizations^22^). Additionally, the report compared 2 or 3 wave performances to highlight development changes over time. Percentiles or norm-referenced scores were given to guide the interpretation of developmental behaviours. Practical advice or recommendations for enhancing the performance were provided only for reference.

#### Brain science popularization

The enrolled participants and their guardians were regularly invited to attend talks popularizing brain science organized by the program staff. The talks were focused on providing an intuitive understanding of the personal development report and promoting extensive knowledge of brain science. During the progress, we emphasized the scientific significance of establishing longitudinal datasets for Chinese children and adolescents, with the aim of encouraging retention in the project. A featured program *Localization of Frontiers for Young Minds articles* (https://kids.frontiersin.org/articles) was launched in July 2019 with weekly neuroscience popularization articles promoted through various social media platforms, such as the *WeChat* Official Accounts Platform. Teenagers volunteered to be part of the translation team and were supervised by the CCNP Science Mentors. This initiative widely popularized background knowledge to school-age students, and improved acceptance of the project among the target population.

### Participant procedure

#### Screening & registration

A prescreening phone interview inquired about each participant’s health history, family history of disease, and any potential risk or side effect associated with the MRI procedure. After a detailed introduction, any necessary explanations and an assessment of those who were willing to participate, individuals meeting inclusion criteria without any reason for exclusion were invited to preregistration. Both participants and their guardians were invited to be confirmed on site, and signed the informed consent form before official participation.

The inclusion criteria were as follows:

- Male or female native Chinese speakers aged 6.0–17.9 years at enrolment. Note that some participants under 6 years old were also enrolled as a preexperiment on younger individuals.
- Must have the capacity to provide assent, guardian must have the capacity to sign informed consent.

The exclusion criteria were as follows:

- Guardians unable to provide developmental and/or biological family histories (e.g., some instances of adoption).
- Serious neurological (specific or focal) disorders.
- History of significant traumatic brain injury.
- History or family history (first-degree relatives) of neuropsychiatric disorders, such as ASD, ADHD, bipolar disorder, or schizophrenia.
- Contraindication for MRI scanning, such as metal implants, or pacemakers.

#### Ethical approval

This project was approved by the Institutional Review Board of the Institute of Psychology, Chinese Academy of Sciences (The Ethical Approval Number: H18017). Prior to conducting the research, written informed consent was obtained from one of the participants’ legal guardians, and written assent was obtained from the participants. Participants who became adults in the longitudinal follow-up provided written consent once becoming 18 years old.

### Experimental design

Detailed assessments are listed in Supplementary Table S1. Data collection was accomplished by well-trained research assistants.

#### Demographics & characteristics

Demographic information (e.g., age, sex and handedness) and characteristics of both participants (e.g., educational level) and their families (e.g., number of children) were collected at the beginning of each wave through self-designed parental questionnaires. The hand preference of the participant was assessed by the Annett Hand Preference Questionnaire (AHPQ)^23^ in the CGK Sample and was classified into 5 subgroups: strong right preference (RR), mixed with right tendencies (MR), mixed (M), mixed with left tendencies (ML), and strong left preference (LL). In the PEK Sample, the Chinese version of Edinburgh Handedness Inventory (EHI)^24^ was applied and participants were classified into 7 subgroups; two additional subgroups compared with the CKG Sample were right preference (R) and left preference (L). Parent-reported Child Behavior Check List (CBCL)^25,26^ was applied with Version: Ages 4–16 (1991 version) in the CKG Sample and Version: Ages 6–18 (2001 version) in PEK Sample. To capture participants’ family characteristics to achieve better population classification, a self-designed parent-reported Subjective Social Status questionnaire using a 10-point self-anchoring scale was additionally conducted in the process of PEK sampling. Additionally, in the PEK Sample, Music Training History Questionnaire for Children^27,28^ was completed by parents to collect information about the participants’ previous training or acquisition of music-related knowledge/skills.

#### Biophysical measures

Objective biophysical measurements include height, weight, head circumference, and biomarkers of cardiovascular health (i.e., blood pressure and heart rate). The blood pressure assessment was performed immediately after the participant’s MRI scan, and the data provided were related to this specific time point. Visual acuity (naked eyesight in general, corrected eyesight as optional if the participant had ametropia) and Pure Tone Audiometry (PTA)^29^ were specifically measured in the PEK Sample. Even though PTA is a relatively basic and important hearing test, and was conducted in a sound-proof room, we note that the results might be affected by other factors, such as the psychological status of the participant. Therefore, we emphasized that the participant’s biophysical characteristics were only related to the physical and emotional state of the moment.

#### Physical fitness measures

Grip strength^30^, standing broad jump^31^ and 15-metre shuttle run^32^ were tested to measure the muscle strength and cardiopulmonary endurance of the participants. After watching the procedure demonstrations, the test method and details were explained to the participants, and they were required to warm up sufficiently. The 15-metre shuttle run was conducted at the end, and the number of completed laps was recorded as the result. Rating of Perceived Exertion (RPE)^33^ was measured immediately after the shuttle run to evaluate exercise intensity.

#### Intelligence quotient measure

All participants aged 6–17.9 were given the Wechsler Intelligence Scale for Children-IV-Chinese Version (WISC-IV)^34^ during each wave’s assessment. Ten core subtests and 4 supplementary subtests were combined to estimate Full Scale Intelligence Quotient (FSIQ) with 4 indices: Verbal Comprehension Index (VCI), Perceptual Reasoning Index (PRI), Working Memory Index (WMI) and Processing Speed Index (PSI). Participants aged above 18 years completed the Chinese Version of Wechsler Adult Intelligence Scale (WAIS-IV)^35^.

#### Psychological behaviour questionnaires

Widely used questionnaires with high reliability and validity, primarily focused on cognition, personality, and issues pertaining to social-emotional functioning (e.g., life events, self-concept, emotions and affects such as stress, anxiety, depression, loneliness, and positive and negative affect) were obtained by one-on-one instruction. All the psychological behaviour questionnaires corresponding to each Sample are detailed in Supplementary Table S1.

#### Psychological behaviour Tasks/Tests

Various experimental paradigms through E-Prime, MATLAB and other platforms were used to assess participants’ cognitive performance in different domains (e.g., executive attention, social cognition, decision-making and language). Some culturally specific tasks were also conducted (e.g., Chinese Character Naming Task). Details are listed in Supplementary Table S1. Before each formal task/test, participants were informed of the overall procedure through an instructional message and allowed to have exercise trials. Brief introductions on these tasks/tests are as follows:

- **Attention Network Test** The classic Attention Network Test (ANT)^36^ was applied to assess the three attention networks: alerting, orienting and executive attention. During the experiment, small cartoon images of “fish” were presented on the centre of the computer screen for a very short time. Participants were asked to determine as soon and as correctly as possible whether the head of the centre “fish” pointed the left or right (we use images of cartoon fish to replace the “arrow” in the classic ANT paradigm for high preference in children and adolescents). Response times (RTs) and accuracy were measured for each trial. Preprocessed outcome variables include accuracy of all trials (%), alerting (*ms*), orienting (*ms*), and control (*ms*). (Note all the details on outcome variables are described in the data supplementary “json” files.)
- **Singleton Stroop Task** This task was introduced to assess an individual’s bottom-up attention capture and top-down inhibitory control^37^. A fixation point was presented on the screen at the beginning and end of each experimental trial. Five short vertical lines were then presented on the screen, and one of the lines was red while the remaining were black. The next task stimulus, a vertical arrow, randomly appeared at the top or bottom of the screen. Participants were asked to respond to the direction of the arrow as quickly and correctly as possible. Preprocessed outcome variables include response time and accuracy of each “congruent” or “incongruent” trail.
- **Task-Switch Paradigm** In this experiment^38^ participants were asked to make judgements as soon and as correctly as possible between two different types of digit categorization: whether the presented digit was greater or less than 5 and whether the present digit was odd or even. RTs and accuracy were measured for each trial. Preprocessed outcome variables include mean reaction time of accuract “repeat” or “switch” trails, and switch cost which represent reaction time difference between switch and repeat.
- **Digit** ***N*-back Task** This paradigm^39^ was used with two levels: 1-back and 2-back. Participants were asked to judge as soon and as correctly as possible whether each stimulus in a sequence, which consisted of nine random digits from 1 to 9, matched the stimulus that appeared *N* items ago. To be specific, participants would determine whether the currently presented digit was the same as the one (i.e., 1-back) or second one (i.e., 2-back) presented before. RTs and accuracy were measured for each trial. Preprocessed outcome variables include accuracy of trails, and mean reaction time of accuract “1-back” or “2-back” trails.
- **Prisoner’s Dilemma** This task was conducted to assess the influence of networks on the emergence of cooperation^40,41^. Before the formal experiment, participants were instructed that there were four blocks of games. Two of them are social partner blocks, in which their partners in each round are peer children and would be paid according to the final outcome. In contrast, in two blocks of nonsocial partner blocks, the partner’s choice was randomly given by computer. In the experiment, first, a fixation point was presented on the screen. Then, a payoff matrix that lists the payoff when the participant and the partner choose to “cooperate” or “betray” is created. Participants were instructed, “You need to choose “cooperate” or “betray” without knowing your partner’s choice. You will then present your partner’s choice and therefore respective benefits based on bilateral choices.” Finally, participants were asked to assess their emotional response towards the choices. Before participating in the social decision-making study, both prisoner’s dilemma and ultimatum game introduced next, the participants were asked to describe themselves in a self-introduction, including their age, upbringing, education, personality, and hobbies. The participants were informed that their self-introduction would be anonymously presented to a group of peers who would participate in the same experiments. Those peers acted as their partners in the experiment. Each of those peer partners independently made a choice after reading the participants’ self-introduction and their choices were preprogrammed in the experiment computer and displayed to them in the experiment.
- **Ultimatum Game** This task was designed to explore whether and how social comparisons with third parties affect individual preferences for fair decision-making^42,43^. Before the formal experiment, participants were told that there are two blocks of games. One of them would be under the “gain” context, which means that players in the game are together to distribute gain. The other would be under the “loss” context, which means that players in the game are together to distribute loss suffering. In the formal experiment, an allocation of gain/loss would be offered, and participants needed to decide to accept or reject the offer and report how satisfied they felt about their final rewards/suffering.
- **Delay Discounting Task** To explore reward evaluation and impulsivity characteristics^44^, in this task, participants were asked to make a series of choices to receive a certain value of fictitious funds immediately, or to wait for a period of time (i.e., a day, a week, a month, three months, or six months) before receiving a larger amount. For example, choosing between “Get ¥100 tomorrow” and “Get ¥50 today”. The reward amount was presented on the screen immediately after each decision was made.
- **Risky Decision Task** This task was designed as an interactive, sequential gambling game to probe the neural correlates of risk taking and risk avoidance during sensation seeking^45,46^. Participants were instructed to play a roulette game with a certain amount principal at the beginning. After deciding whether to participate in the gamble or not depending on the situation introduced (the odds of winning the jeton), rewards (gain or loss) were presented. Each decision had to be made in 4 seconds. After each trial participants were asked to evaluate and report whether they had made the right choice.
- **Chinese Character Reading Test: Chinese Character Naming Task** This task was introduced to examine children’s reading ability and to determine potential developmental issues in the process of reading acquisition^47^. Participants under 12 years old were asked to read a list of 150 Chinese characters (increasing difficulty from front to back) one by one. The score was calculated from the number of characters reading correctly.
- **Lexical Identification** This task used the semantic priming paradigm to examine mental representations of word meanings and their relationships^48,49^. Critical words consisted of real word targets following a thematic prime (e.g., eat-lunch) or a categorical prime (e.g., apple-banana). Additionally, filler words consisting of nonword targets (e.g., eat-unch) were also added. The words (both the prime and target words) were consecutively presented on the screen and after the presentation of each word, participants were asked to judge whether the word was a real word or not. RTs and accuracy were recorded.
- **Audiovisual Integration of Words** This task examined the integration of visual and auditory word information^50,51^. In each trial, participants were visually presented with one Chinese character on the screen and presented with a word pronunciation at the same time. The character was either audiovisually congruent (where the character and the pronunciation were matching) or incongruent (where the character and the pronunciation were nonmatching). Participants were instructed to judge whether the auditory word pronunciation matched the visual word form. RTs and accuracy were recorded.
- **Brief Affect Recognition Test** This test was used to evaluate an individual’s recognition of facial expressions^52,53^. Participants were first presented with a 200 *ms* fixation point in the centre of the screen, and then randomly presented with a picture of a model’s emotional expression for 200 *ms*. Ten models (six women and four men) were selected from the Ekman database. Participants were asked to judge the expression presented from two options within the limited time (200 *ms*), or they would automatically skip to the next image. Failed to select an expression was marked as wrong. There were 30 sets of facial expressions made up of six different facial emotions (happiness, sadness, fear, disgust, surprise, and anger).
- **Temporal Bisection Paradigm** To evaluate an individual’s characteristics on time perception^54,55^, participants were required to learn two time intervals to strengthen their memory of long and short time intervals. These time intervals were defined as the presenting a 2 *cm* × 2 *cm* black squares was presented. For short duration, the black squares were presented for 400 *ms*, for long duration, the black squares were presented for 1600*ms*. After the training procedure, participants were instructed to judge the length of the test time intervals (rating intervals as “long” or “short”) according to the previously learned time intervals. Black squares were randomly presented 20 times for 400,600,800,1000,1200,1400, or 1600*ms* interval.
- **Ebbinghaus Illusion** To assess participants’ susceptibility to perceptual illusions^56,57^, participants were instructed to view a screen with a grey background. A probe circle and a reference circle were presented on the left and right sides of the central fixation point. The probe circle was always surrounded by a group of smaller circles. The reference circle, which was fixed in size, was surrounded by larger circles. The perceptual sizes of the probe circle and reference circle were not the same. The task was to adjust the size of the probe circle with up or down arrow key to match that of the reference circle. A chinrest was used to help minimize head movement. The illusion size was measured by (size of test circle - size of reference circle)/size of reference circle.
- **Binocular Rivalry** To evaluate sensory eye dominance^58,59^, participants were instructed to view two orthogonal sinewave grating disks (±45° from vertical) dichoptically through a pair of shutter Goggles (NVIDIA 3D Vision2 glasses). A chinrest was used to minimize head motion. The gratings were displayed in the centre of the visual field and were surrounded by a checkerboard frame that promoted stable binocular alignment. Participants were required to report whether they perceived one of the two gratings or the mix of them by holding down one of the three keys (Left, Right, or Down arrows) on the keyboard. If a key was not pressed within a predetermined period of time, there would be an audible alarm for the participants.
- **Ocular-tracking Task** This task examines basic visuomotor ability by measuring ocular-tracking performance, as previously described^60–64^. This task was based on the classic Rashbass step-ramp paradigm^65^ modified to accommodate a random sampling of the polar angles from 2° to 358° in 4° increments around the clock face without replacement using 90 trials. Each trial began with a cartoon character (Donald Duck or Daisy, 0.64°*H* × 0.64°*V*) in the centre of a black background on a computer screen. Participants were asked to fixate on the central character and initiated the trial by pressing a mouse button. After a random delay drawn from a truncated exponential distribution (mean: 700 *ms*; minimum: 200 *ms*; maximum: 5,000 *ms*), the character would jump in the range of 3.2° to 4.8° away from the fixation point and immediately move back at a constant speed randomly sampled from 16°/*s* to 24°/*s* towards the centre of the screen and then onwards for a random amount of time from 700 to 1,000 *ms* before disappearing. To minimize the likelihood of an initial catch-up saccade, the character always crossed the centre of the screen at 200 *ms* after its motion onset. Both the character speed and moving direction were randomly sampled to minimize expectation effects. Participants were instructed to keep their eyes on the character without blinking once they initiated the trial and then to use their eyes to track the character’s motion as best as they could until it disappeared on the screen. Preprocessed outcome variables include latency, open-loop acceleration, steady-state gain, proportion smooth, saccadic rate, saccadic amplitude, saccadic precision, eye response precision, anisotropy, asymmetry, speed noise and responsiveness.
- **Dichotic Digit Test** This test was used to assess individuals’ binaural integration^66,67^, attention allocation, and auditory/speech working memory ability. A different set of digits (2 or 3 digits) was presented simultaneously to the participant’s left and right ears with an output intensity set to 50 *dB* HL. Participants were asked to listen carefully and repeat the digits heard from right ear to left ear during half of the trials, and from left ear to right ear during the other half of the trials. The orders were counterbalanced between participants. Preprocessed outcome variables include accuracy of four trials in reported in either left of right ear.
- **Competing Sentences** This test was introduced to examine auditory selective attention and the ability to inhibit irrelevant utterance interference during speech recognition^67^. Two simple Chinese sentences with the same syntactic structure but different contents (7 words with 4 key words, e.g., “the turtle/swims slower/than/the whale” (“乌龟/比/鲸鱼/游得慢”)) were presented simultaneously to the left and right ears. Participants were asked to listen carefully and repeat the content in the attended ear (i.e., the output intensity of the attended ear was 35 *dB* HL while the nonattended side was 50 *dB* HL) at the end of the sentence. The attended ear was left on half of the trial and right on the other half. The orders were counterbalanced between participants. Preprocessed outcome variables include averaged accuracy for each ear.
- **Mandarin Hearing in Noise Test for Children** This test was used to assess speech recognition ability in a noisy environment^68^. A simple Chinese target sentence (15 sentences of 10 Chinese syllables each, e.g., “He drew a tiger with a brush” (“他用画笔画了一只老虎”)) was presented by the frontal speaker, and speech spectrum noise was simultaneously played by the frontal or lateral (90° apart) speaker. The noise intensity was constant at 65 *dB* SPL, and the starting signal-to-noise ratio (SNR) was 0 dB for the front noise speaker and −5 dB for the side noise speaker. Participants were required to listen carefully and repeat the sentence at the end. The SNR threshold at which participants correctly reported 50% of syllables in the sentence was recorded as the speech recognition threshold (SRT).
- **Verbal Fluency** The verbal fluency test was used to evaluate strategic search and retrieval processes from the lexicon and semantic memory^69,70^. Participants in each trial were required to speak nonrepeated words based on one given category within 1 minute. There were two trials for semantic fluency and two for phonemic fluency. Semantic fluency required participants to say as many words as possible belonging to a particular semantic category (fruit, animal). Phonemic fluency required the participants to say as many different words as possible (excluding proper names) beginning with a Mandarin initial consonant (/d/ and /y/) but not repeating the first vowel and tone. Preprocessed outcome variables include the number of unique answers belonging to each trail. The last four behaviour tests were performed in a soundproof room in one session, and normal hearing in both ears (average hearing threshold ≤20 *dB* HL from 250 to 8000 *Hz*) was required.

#### MRI mock scan

In the preparation stage for MRI scans during PEK sampling, mock scanning was performed to improve participant compliance by alleviating anxiety and psychological distress, and to facilitate the success of scans, especially for participants under 12 years old (i.e., primary education stage)^71^. The mock scanner room was built in a child-friendly atmosphere (e.g., child-style decorations, toys or books for different ages, etc) which provided a relaxed buffer zone. A real-size mock scanner built by PST (Psychology Software Tools, Inc.) using a 1:1 model of the GE MR750 3 T MRI scanner in use at the PEK site, allowed participants an experience faithful to the actual MRI scanning procedure. Participants were guided to lie still on the bed listening to the recorded MRI scanning sounds and watching the screen through the mirror attached to the model head coil. Three imaging scenarios were performed: resting-state fMRI (rfMRI), morphometric MRI and natural stimulus fMRI (ns-fMRI) which refers to the movie-watching state in this sample. Each scenario lasted at least five and a half minutes. The instructions were consistent with the actual MRI scan, except the movie clip played during the natural stimulus was replaced by additional resources. Head motion data were automatically acquired with the MoTrack Head Motion Tracking System (PST-100722).

#### Magnetic resonance imaging

MRI data of CKG Sample were collected using a 3.0-T Siemens Trio MRI scanner (sequencing order: rfMRI→T1-weighted→rfMRI→T2-tse/tirm) at the Center for Brain Imaging, Southwest University. The PEK Sample were imaged on a 3.0-T GE Discovery MR750 scanner at the Magnetic Resonance Imaging Research Center of the Institute of Psychology, Chinese Academy of Sciences (sequencing order: rfMRI→T1-weighted→rfMRI→T2-weighted→ns-fMRI→DTI). Imaging sequences remained the same across all waves at each site but were different between the two sites and optimized for similar space and time resolutions. Minimal adjustments to sequencing order would occur as necessary. To avoid introducing cognitive content or emotional states into the resting-state condition, the rfMRI scans were always conducted before movie-watching. The detailed acquisition parameters in both samples are presented in Table 2. T2 imaging details are not listed here as it usually used for detecting organic brain disease, which is a complementary setup in our data collection enrolling typically developing participants only. MRI procedure was performed within one session, small breaks were allowed and instructions were given before starting each sequence. During data collection there were no software or hardware upgrades that would affect the MRI scanning performance.

- **Resting-state fMRI** Two rfMRI scans with identical (within each Sample) parameters were acquired and separated by a T1-weighted sequence. Participants were asked to keep their eyes fixated on a light crosshair (CKG Sample) or a cartoon image (PEK Sample) on the dark screen, to stay still, and not to think of anything in particular. Noise-cancelling headphones (OptoACTIVE^TM^ Active Noise Control Optical MRI Communication System, Version 3.0) were provided in the PEK Sample rfMRI scan to foster a more comfortable imaging experience.
- **Morphometric MRI** Morphometric imaging consisted of T1-weighted, T2-weighted (PEK Sample only) and T2-tse/tirm (CKG Sample only) scans. A T2 scan was performed after two rfMRI scans to evaluate brain lesions and improve cross-registration. For both morphometric scans, participants were asked to keep their eyes closed to rest.
- **Natural Stimulus fMRI** This functional MRI condition was implemented in the PEK Sample only and under a movie-watching state. Movie watching mimics real-world experiences related to the context. Movie watching requires the viewer to constantly integrate perceptual and cognitive processing. Movie-watching helps to reduce head motion and increase participant compliance and, therefore, improve the feasibility of brain-behaviour association studies^72^. At the beginning of PEK sampling, participants were watching an audiovisual movie clip consisted of 3 segments, a clip from movie “Zootopia”(Chinese dubbing), advertisement “Taxi” from Tesco Lotus and “My Dad is a Liar” from MetLife. From August 2020, the movie clip was replaced by an animated film named “Despicable Me”^73^ (6 *m*:06 *s* clip, DVD version exact times 1:02:09–1:08:15, spanning from the bedtime scene to the getting in a car scene).
- **Diffusion Tensor MRI** This sequence was implemented in the PEK Sample only. During the scans participants were free to decide if they wanted to watch another animation clip or rest. Detailed parameters are presented in Table 2.

**Table 2:** MRI Protocol Parameters.

	CKG Sample	PEK Sample	
**Scanner**	**Siemens Trio Tim, 3.0 T**	**GE Discovery MR750, 3.0 T**	
**Head Coil**	**12-channel**	**8-channel**	
	**r-fMRI**	**r-fMRI**	**ns-fMRI**
Sequence	EPI	Gradient Echo	Gradient Echo
Time Acquisition (min:sec)	7 m:45 s	6 m	6 m:06 s
Slices	38	33	33
% FOV Phase	1	1	1
Resolution (mm)	3 × 3 × 3	3.5 × 3.5 × 4.2	3.5 × 3.5 × 4.2
matrix	72 × 72	64 × 64	64 × 64
TR (ms)	2500	2000	2000
TE (ms)	30	30	30
Flip Angle (°)	80	90	90
Notes			
	**T1-weighted**	**T1-weighted**	**DTI**
Sequence	3D MPRAGE	3D SPGR	Spin Echo
Time Acquisition (min:sec)	8 m:19 s	4 m:41 s	10 m:54 s
Slices	176	176	75
% FOV Phase	1	1	1
Resolution (mm)	1 × 1 × 1	1 × 1 × 1	2 × 2 × 2
matrix	256 × 256	256 × 256	112 × 112
TR (ms)	2600	6.7	8724
TE (ms)	3.02	2.9	81.4
Flip Angle (°)	8	12	90
Notes	900 ms TI	450 ms TI	64 directions, 10 b0 images, b = 1000 s/mm^2^

### Summarizing lessons learned

Throughout the implementation of the pilot devCCNP, we faced several challenges and gained valuable insights. We are continuing to improve strategies in dissemination, recruitment, retention, and characterization. Here are some key considerations that may aid similar endeavours, including large-scale sampling projects (e.g. the national longitudinal cohort on child brain development in China).

#### Recruitment strategy

Due to the particularity of children and adolescents, studies involving this population typically encounter significant challenges. All projects should be conducted on the premise of not affecting academic progress and ensuring safety. Both school- and community-based recruitment have distinct advantages and, inevitably, inherent drawbacks.

- **School-based Strategy** Support of flexible schoolwork arrangements matched with the sampling schedule can greatly ensure the quantity and quality of data collection for junior and senior high school participants. With the help and encouragement from coordinators in school, recruitment efforts could be reduced. However, for these same reasons, the participants’ motivation could be compromised, as they may not be primarily driven by their interest in the project or may lack a clear understanding of the value and the contribution of participation. Meanwhile selecting recruiting schools may also, to some extent, reduce the sample representativeness of the target population.
- **Community-based Strategy** Younger participants are undoubtedly easier to recruit in the community, but the number of pubertal-age participants is limited especially for longitudinal studies. Self-enrolled participants recruited at the community level or their guardian typically possess relevant knowledge and understand the value of participating in the project; therefore they are strongly motivated and tend to cooperate better. However, this also biases the sample to families with higher levels of education, or with some uncertain developmental problems. Especially with word of mouth spreading and popularizing, the similarity between participants’ families (e.g., social status, economic background) and/or their characteristics would be higher, which might diminish the individual differences between the participants.

The drawbacks outlined above can be compensated by combining diverse recruitment strategies and expanding the age range and geographical regions of the recruitment. This approach could enable a greater diversity of physical, psychological and cognitive phenotypes and promote the establishment of a typical developing cohort.

#### Experimental design

Charting the typical developmental trajectories of individuals (with respect to physical, psychological and morphological development) through longitudinal design greatly contributes to uncovering the complex relationship between the brain and behaviour. As a long-term project, it is important not only to assess the full range of participants’ current state at a single time point of data collection, but also to capture what important life or social events occur during the follow-up period. These events include, but are not limited to, a family event (e.g., death of a family member, divorce of guardians), the birth of siblings, sudden illness, a significant social or public health event, and others. Future projects could employ regular questionnaires or scales (e.g., monthly) to collect related information during follow-up intervals, so that relevant details could be recorded. Alternatively, participants could be asked to retrospectively report the events at each time point of data collection, but this may miss the ability to capture their physiological or psychological experiences at the time of the event.

#### Practical experience

The attentiveness and compliance of participants have significant impacts on data quality. The following lists the lessons we have learned in the course of our practice.

- **Questionnaire and Scale** For large-scale projects involving different economic or cultural areas (e.g., northern and southern China), it is recommended to apply both questionnaires or scales consisting of subjective and objective assessments. For example, it is suggested to apply Subjective Social Status and to inquire about family income to assess participants’ family economic status. The combination of objective and subjective questions for the same evaluation purpose can better classify populations living in areas of significant cultural differences. This recommendation also applies to other physical, psychological and cognitive assessments.
- **Behaviour Tasks/Tests** Most of the behavioural measurements tested with computers require convenient interactions with participants. Tasks requiring participants to press keys quickly is not conducive to young children if the keys are too small or placed too close. For example, pressing “1” or “2” on a keyboard is more likely to cause errors than pressing “A” or “M”. Some measurements have higher requirements with respect to participant posture (e.g., ocular-tracking task requires the participant to operate the mouse while keeping the head and upper body still). Therefore, the number of trials and the duration of each trial need to be carefully designed. An overall time of less than 20 minutes for completion is recommended for young participants. At the same time the related hardware equipment should be able to accommodate a broad range of participant characteristics (e.g., head circumference, height, bodily form). For example, common ocular-tracking devices in the laboratory need to be equipped with stable chairs that can be adjusted for a wide range of heights, child-sized desks, or chinrests that can restrain the head. It is recommended to invite children of each age group to evaluate all the experimental protocols at the design stage.
- **Magnetic Resonance Imaging** A scanning time for one MRI session of no longer than 45 minutes (one hour maximum) is strongly recommended, especially for junior participants. In prticular, mock scan training before formal MRI was shown to effectively improve the success of imaging. In general, training immediately before the formal MRI can be effective although additional mock training episodes before the formal MRI day could also be considered if the participants are particularly scared, are sensitive to sound, or find it difficult to concentrate.
- **Personalized Schedule** During each wave’s data collection, as shown in Table 1, the order in which the tests are scheduled needs to be thoroughly arranged. To better achieve MRI data collection in this project, in principle, MRI was arranged at the beginning of each wave. For those who had more than 2 visits within one wave, IQ measurements were scheduled on the last visit, as they were usually of greater interest to guardians. Physical fitness tests should not be scheduled within a few hours before MRI scans; measurements concerning visual perception should not be scheduled after the measurement that require staring at digital screen for an extended duration.
- **Implementation Progress** Generally, one-on-one instruction from the same implementer across visits (within or even across waves) can be conducive to friendly and cooperative relationships with the participants and can be, especially helpful in relieving the timidity of young children to strangers. Measurements with higher qualification requirements for the implementer (i.e., IQ measure) are recommended to be conducted by limited authorized staff. It is worth mentioning that, unless it is ethically required, we do not recommended that parents be allowed to observe the participant’s engagement process, as this may have the potential to impact their child’s performance.

## Data Records

### Dataset deposition

The devCCNP data has been publicly shared in the *Chinese Color Nest Project (CCNP) – Lifespan Brain-Mind Development Data Community* (https://ccnp.scidb.cn), which is a public platform supported by the National Science Data Bank (https://www.scidb.cn/en) for sharing CCNP-related data and promoting the cooperation of open neuroscience. To offer a better data acquisition, we only upload the MRI dataset on this platform, all the phenotypic data are sharing via deepneuro@bnu.edu.cn once the users’ data access applications are approved by the Chinese Color Nest Consortium (CCNC).

#### devCCNP Full

This release contains the full measurements of devCCNP protocol. MRI data have been deposited into the Science Data Bank^74^ (10.57760/sciencedb.07478). The full dataset will be accessible upon requests submitted according to the instructions described below. A sample of the longitudinal data from a participant is fully accessible through FigShare (10.6084/m9.figshare.22323691.v1) to demonstrate the data structure^75^. Note that T2-tes/tirm in CKG Sample, which has only 30 slices used for detecting organic brain disease, was not uploaded. As it is not frequently used in scientific research. Any application for this part of data would be transferred case-by-case.

#### devCCNP Lite

This release version contains only basic demographics (sex, age and handedness), T1-weighted MRI, rfMRI and diffusion tensor MRI data of devCCNP. No cognitive or behavioural information is included. The devCCNP Lite will be accessible upon the requests according to the instructions described below. Data have been deposited into the Science Data Bank^76^ (10.57760/sciencedb.07860).

### Data structures

**Fig. 2:**
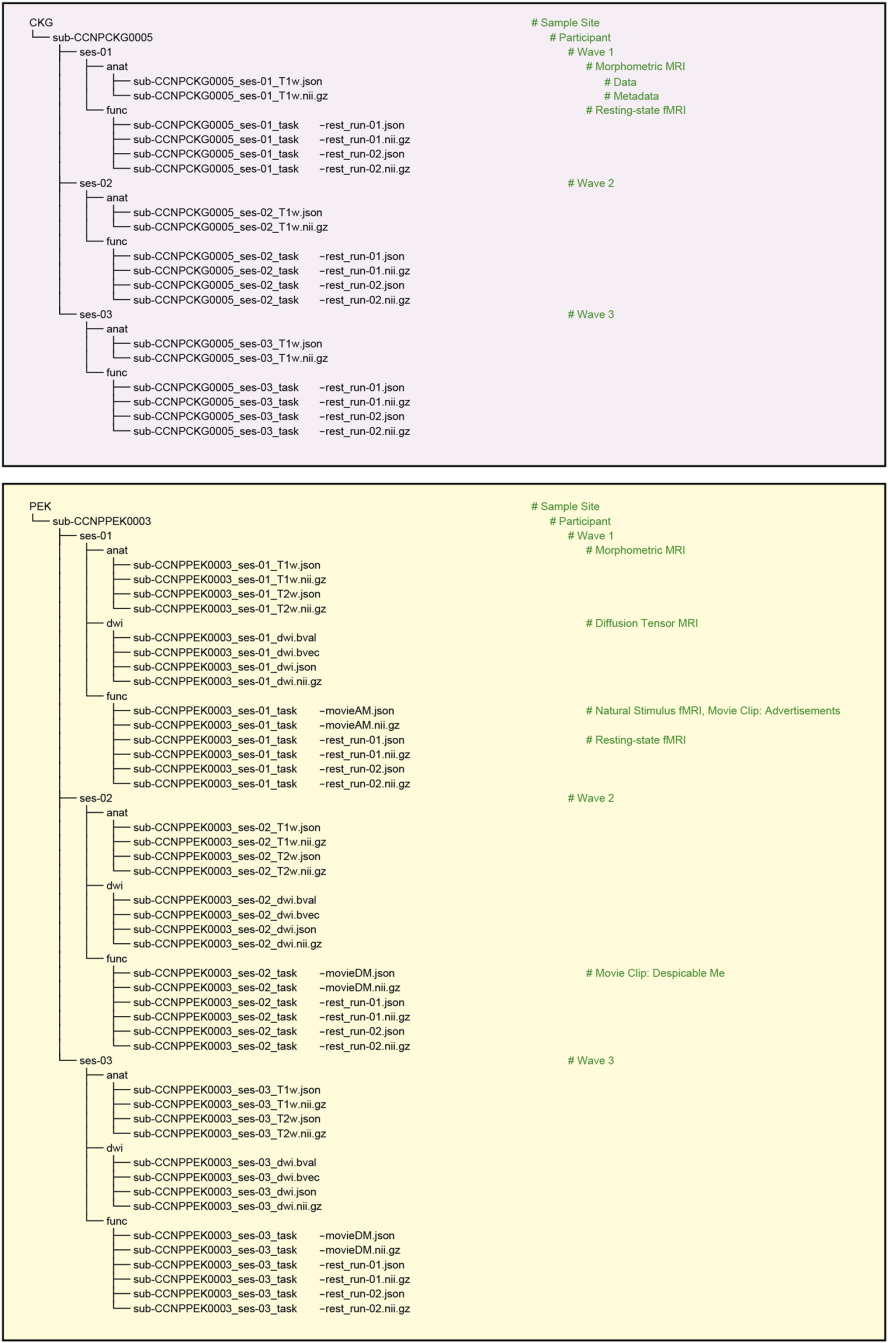
Example of the MRI raw data directory structure. Collected MRI raw data are structured within a hierarchy of folders according to the standard BIDS format. Under the toplevel project folder “devCCNP/”, CKG (top) and PEK (bottom) Samples are organized separately. Each participant’s folder “sub-CCNP*/” may contain several subfolders depending on how many waves have been completed to date (i.e., if all waves are completed, the folder would include three “/ses-*” subfolders). Imaging data (“.nii.gz”) and metadata (“.json”) are organized into modality-specific directories “/anat/”, “/func/” and “/dwi/”. Note that in the PEK Sample, Diffusion Tension Imaging (DTI) data files are stored under “/dwi/” folders with datatype name “*_dwi.*”.

All data files are organized according to the Brain Imaging Directory Structure (BIDS) standards^21^. An example of the MRI data storage structure is presented in Fig. 2. Under the top-level project folder “devCCNP/“, CKG and PEK Sample are organized separately. Each participant’s folder “sub-CCNP*/” may contain several subfolders depending on how many waves have been completed to date (i.e., if all waves are finished, the folder would include three “/ses-*” subfolders). Imaging data (“.nii.gz”) and metadata (“.json”) are organized into modality-specific directories “/anat/”, “/func/” and “/dwi/”. Note that in the PEK Sample, Diffusion Tension Imaging (DTI) data files are stored under “/dwi/” folders with the datatype name “*_dwi.*“. All demographic and behavioural data are structured under the “/beh/” folder (“.tsv”). Detailed parameters of each psychological behaviour task/test are provided in the “json” file attached.

### Partial and missing data

Some participants were not able to complete all components of the CCNP protocol due to a variety of situations (e.g., delay or cancel caused by the COVID-19 pandemic). Overall, we logged data collection if any issues occurred that required extra attention during analysis (see details written in the “json” file attached to each data).

### Data licence

To access data, investigators must complete the application file *Data Use Agreement on Chinese Color Nest Project* (DUA-CCNP) located at: http://deepneuro.bnu.edu.cn/?p=163 and have it reviewed and approved by CCNC. Compliance with all terms specified by the DUA-CCNP is required. Meanwhile, the baseline CKG Sample on brain imaging is available to researchers via the International Data-sharing Neuroimaging Initiative (INDI) through the Consortium for Reliability and Reproducibility (CoRR)^77^. More information about CCNP can be found at: http://deepneuro.bnu.edu.cn/?p=163 or https://github.com/zuoxinian/CCNP. Requests for further information and collaboration are encouraged and considered by CCNC; please read the Data Use Agreement and contact us via deepneuro@bnu.edu.cn.

## Technical Validation

### Sample composition

A total of 479 participants completed baseline visits, 247 (51.6%) completed the second wave data collection, and 138 (28.9%) completed the third wave (i.e., final protocol) as of December 2022. There were 648 (75.0%) measurements completed by participants twelve years old or younger. The number of participants who completed visits in each age cohort are shown in Table 4, and age and sex composition are presented in Fig. 1c,d. Demographic and enrolment data for both the CKG Sample (enrolled in 2013–2017) and the PEK (enrolled in 2018–2022) Sample are listed in Table 5. As mentioned above, the overall design has a longitudinal follow-up interval of 15 months, to which the CKG Sample consistently adhered; however, during the PEK sampling, the intervals were prolonged. For instance, inevitable practical situations affected community-based recruitment, primarily the COVID-19 pandemic. Please note that during COVID-19, data collection was suspended from January to August 2020. We designed questionnaires to assess participants’ learning and daily life status^12^. Each participant’s sampling age and corresponding intervals are presented in Fig. 1e–h. For all of the measurement intervals, 122 (32.1%) were achieved by design.

**Table 3:** Distribution and statistics of IQ measures.

IQ Measures	Sample	Median	Mean	SD	Shapiro–Wilk Test		Rank-Sum Test	
					W-value	P-value	W-value	P-value
FSIQ	CKG	111	111.54	12.08	0.99	0.01	99068.50	0.00
	PEK	121	120.58	15.49	1.00	0.68		
	devCCNP	115	115.52	14.39	0.99	0.00	/	
PSI	CKG	104	106.71	14.00	0.96	0.00	71633.00	0.92
	PEK	104	106.79	15.52	0.98	0.00		
	devCCNP	104	106.75	14.67	0.97	0.00	/	
WMI	CKG	97	98.90	10.85	0.97	0.00	105471.00	0.00
	PEK	109	110.11	14.37	0.99	0.02		
	devCCNP	103	103.77	13.67	0.98	0.00	/	
PRI	CKG	106	107.85	12.49	0.99	0.01	100167.00	0.00
	PEK	118	117.69	15.30	0.99	0.00		
	devCCNP	112	112.12	14.61	0.99	0.00	/	
VCI	CKG	118	118.47	15.48	0.98	0.00	82407.50	0.00
	PEK	122	123.34	18.56	0.99	0.01		
	devCCNP	120	120.59	17.05	0.99	0.00	/	

^1^Median, mean and standard deviation of FSIQ and each of four indices. Shapiro-Wilk test of median values suggests that the sample data commonly disobey a Gaussian distribution, except FSIQ of PEK Sample. Between two sites, Rank-Sum test of each indices shows only PSI has no significant difference. FSIQ, Full Scale Intelligence Quotient; PSI, Processing Speed Index; WMI, Working Memory Index; PRI, Perceptual Reasoning Index; VCI, Verbal Comprehension Index.

**Table 4:** Enrollments of each age cohort in two Samples.

Age Group	Sample	≤5	6	7	8	9	10	11	12	13	14	15	16	17	≥18	Enrollments
Wave1	CKG	0	7	21	19	22	27	29	10	20	10	10	20	12	0	207
	PEK	8	37	51	31	47	36	24	16	5	4	4	3	3	3	272
Wave2	CKG	0	0	2	17	18	24	20	30	7	14	9	7	9	6	163
	PEK	0	0	7	12	15	13	17	10	4	4	1	0	0	1	84
Wave3	CKG	0	0	0	0	8	25	15	6	18	7	7	7	2	6	101
	PEK	0	0	0	1	2	10	3	8	4	3	4	2	0	0	37
Total		8	44	81	80	112	135	108	80	58	42	35	39	26	15	864

**Table 5:** Enrollment profile at two Samples.

	CKG Sample	PEK Sample	Total
**Site**	Southwest University	Chinese Academy of Sciences	
**Location**	Beibei District, Chongqing	Chaoyang District, Beijing	
Area	Southwest China	North China	
Sample Environment	School-based	Community-based	
**Enrollment**	207	272	479
Female	109	121	230
Male	98	151	249
Under 12 Years Old	125	234	359

### Quality assessment

#### Phenotypic data

**Fig. 3:**
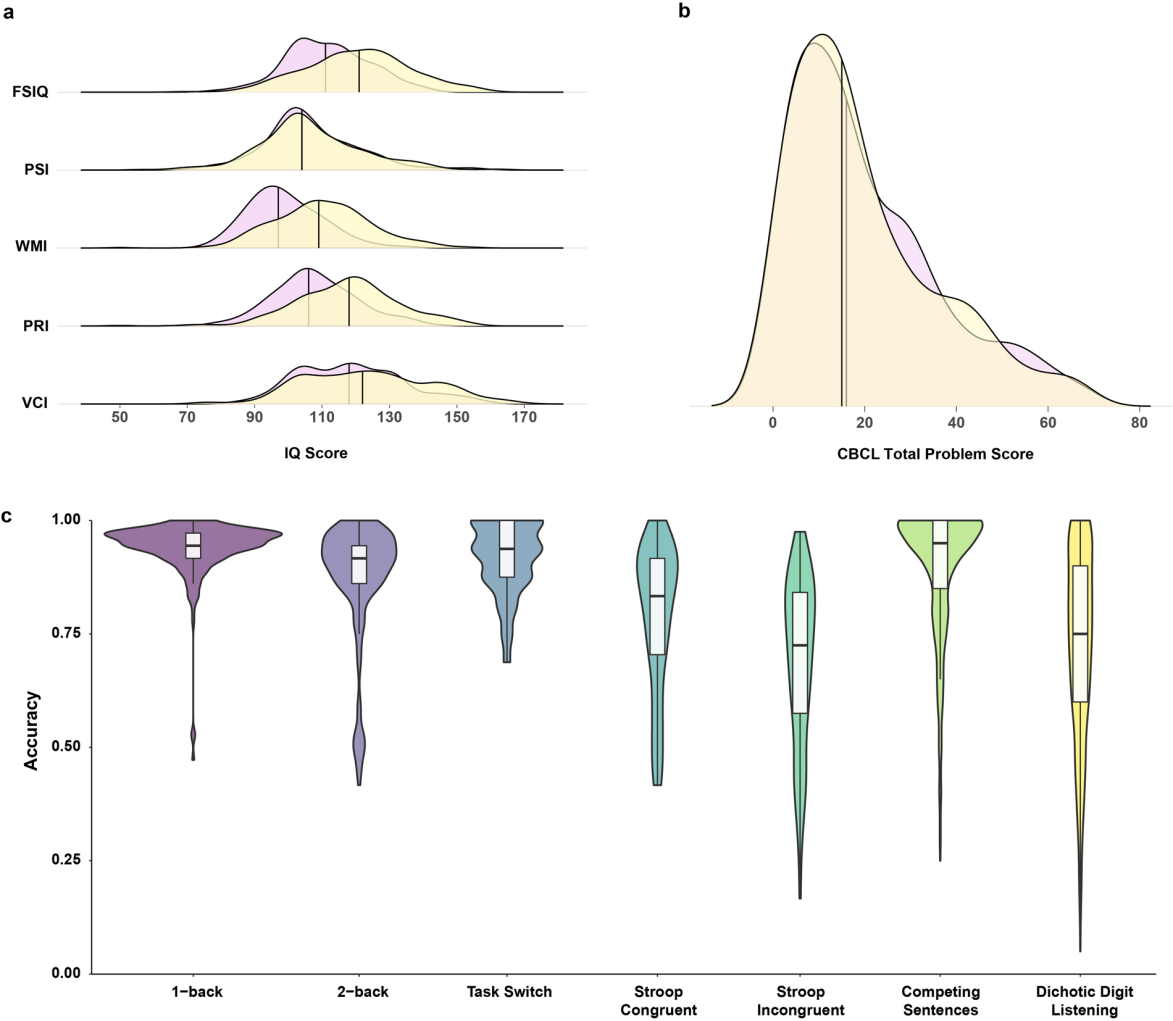
Example of performance on several core characterization measures. (**a**) Distribution of Full Scale Intelligence Quotient (FSIQ) with four indices: Processing Speed Index (PSI), Working Memory Index (WMI), Perceptual Reasoning Index (PRI), and Verbal Comprehension Index (VCI). Related statistical results are shown in Table 3. (**b**) Distribution of CBCL total problem scores. Two samples are displayed separately (CKG, light pink; PEK, canary) in (**a,b**), and vertical lines indicate the medians of samples. (**c**) Distribution of accuracy rates for seven behaviour measurements. Extremely low values are removed for plotting. Data are represented for measurements of all waves.

All of the psychological and behavioural data were made available to users regardless of data quality. We provided all the information on situations that may affect the quality of data within the “json” file. This can guide investigators decisions regarding inclusion of the result data. To verify whether the measured distributions obey a normal distribution, we performed preliminary statistical analysis of several core behaviour measures in the dataset (Fig. 3). Distributions of FSIQ and four indices are shown in Fig. 3a. We summarize the median, mean and standard deviation for each Sample. As shown in Table 3, the Shapiro-Wilk test suggests that the sample data commonly disobey a Gaussian distribution. We believe that this is a common situation that arises when recruiting from the local community (PEK sample), as the program tends to attract parents with high levels of education who place greater emphasis on education. Better education conditions could result in higher IQ. Furthermore, the IQ scores were normalized based on the normative model of Chinese children established in 2008^34^, which may be out of time. Additionally, there was a significant difference between the FSIQ, WMI, PRI and VCI performance of the two samples as identified by the rank-sum test. Mental health assessed by CBCL scores demonstrated that the majority of participants were in the normal range (Fig. 3b) with only 12 participants (1.77%) exhibiting CBCL total problem scores ≥70. We performed preliminary statistical analysis of several common-used cognitive and behavioural measures and present their accuracy rates in Fig. 3c.

#### Structural MR imaging

Structural MRI images were first anonymized to remove all facial information from the raw MRI data. We obscured the facial information using the face-masking tool customized with the Chinese paediatric templates^16^. The anonymized images were then denoised by spatially adaptive nonlocal means and corrected for intensity normalization in the Connectome Computation System (CCS)^78^. To extract individual brains, we trained a deep learning method using a small set of semiautomatically extracted brains in the CKG Sample, and then applied it to all the devCCNP samples. The preprocessed brain volumes were all in the native space and fed into the FreeSurfer (version 6.0) pipeline to obtain general morphological measurements of different brain morphometry. All the preprocessing are accomplished through Connectome Computation System (module H1), scripts can be found at github (https://github.com/zuoxinian/CCS/tree/master/H1). We visually inspected the quality of the T1-weighted images, and two raters were trained to rate the quality using a 3-class framework^79^, with “0” denoting images that suffered from gross artefacts and were considered unusable, “1” with some artefacts, but that were still considered usable, and “2” free from visible artefacts. Images with an average score lower than “2” across the two raters were excluded. A total of 761 (91.9%) images passed the quality control, with 436 (94.8%) images in the CKG Sample and 325 (88.3%) images in the PEK Sample. The intra-class correlation coefficient of the two raters was 0.532.

#### Functional MR imaging

Resting state fMRI(rs-fMRI) data preprocessing^78^ included the following steps: (1) dropping the first 10 *s* (5 TRs) for the equilibrium of the magnetic field; (2) correcting head motion; (3) slice timing; (4) despiking for the time series; (5) estimating head motion parameters; (6) aligning functional images to high resolution T1 images using boundary-based registration; (7) mitigating nuisance effects such as ICA-AROMA-derived, CSF and white matter signals; (8) removing linear and quadratic trends of the time series; (9) projecting volumetric time series to *fsaverage5* cortical surface space; and (10) 6-mm spatial smoothing (we also provide a version of preprocessing results without smoothing). All preprocessing scripts of the above steps are available on github (https://github.com/zuoxinian/CCS/tree/master/H1)^78^. Scans with a mean FD greater than 0.5 were excluded. A total of 452 (98.3%) scans in the CKG Sample and 328 (92.4%) scans in the PEK Sample had at least one rfMRI passed the quality control in each session.

### Brain growth charts

Growth charts on height, weight and head circumference are a cornerstone of paediatric health care. A similar tool has been recently generated for lifespan development of human brain morphology^18^ by LBCC (https://github.com/brainchart/lifespan). While promising for characterizing the neurodevelopmental milestones and neuropsychiatric disorders^18,80^, these charts need more diverse samples to enhance their utility in practice^81^. Here, we employed the devCCNP Sample and the NKI-Rockland Sample (NKI-RS) for Longitudinal Discovery of Brain Development Trajectories^82^ to upgrade the LBCC charts. All the preprocessed T1-weighted MRI images from devCCNP and NKI-RS were subjected to the same manual quality control procedure from the same raters at each site.

Specifically, the maximum likelihood method was used to estimate sample-specific or site-specific statistical offsets (random effects, i.e., mean *μ*, variance *σ*, and skewness $\upsilon$) from the age- and sex-appropriate epoch of the normative brain growth trajectory modelling through the Generalized Additive Models for Location, Scale and Shape (GAMLSS: see details of the site-specific growth chart modeling in Fig. 5 from the LBCC original work^18^). Out-of-sample centile scores for each participant from the devCCNP and the NKI-RS site benchmarked against the offset trajectory were estimated. The normative growth trajectories were estimated for not only global neurotypes including total cortical grey matter volume (GMV), total white matter volume (WMV), total subcortical grey matter volume (sGMV), global mean cortical thickness (CT) and total surface area (SA) but also regional neurotypes, including the summation volumes of the corresponding 34 cortical areas of the two hemispheres, according to the Desikon-Killiany (DK) parcellation^83^.

**Fig. 4:**
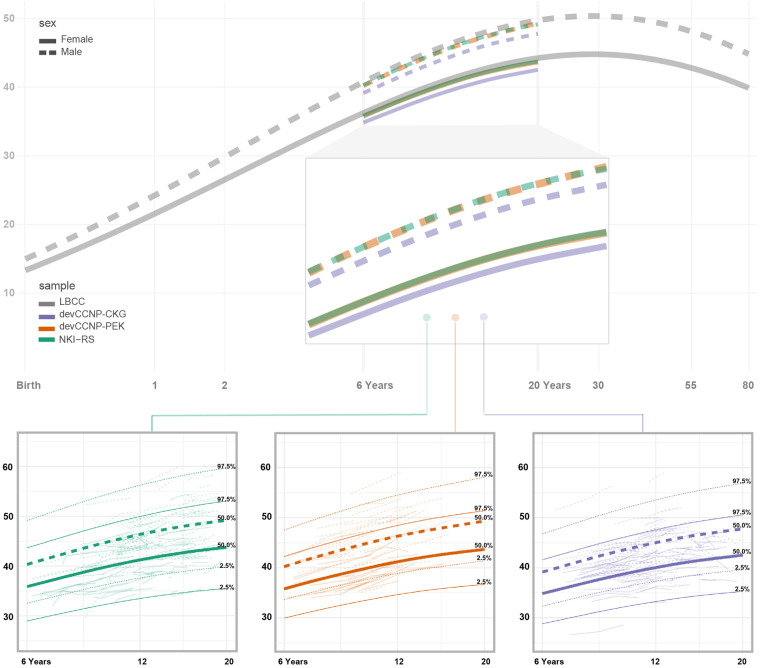
Site/sex-specific brain charts of white matter volume (WMV). The sex-specific lifespan brain charts of WMV (LBCC, light gray) were adjusted by leveraging the school-aged (6–18 years old) samples for three sites (devCCNP-CKG, purple; devCCNP-PEK, orange; NKI-RS, green). The site-specific brain charts are depicted with their percentiles (2.5%, 50%, 97.5%) for males (dashed lines) and females (solid lines). The background polylines characterize individual WMV changes (unit: 10 *ml* or 10,000*mm*^3^) extracted from the multicohort accelerated longitudinal samples.

According to the lifespan WMV trajectory from the LBCC seminal work^18^ (i.e., rapid growth from mid-gestation to early childhood, peaking in young adulthood at 28.7 years), we presented the growth curves of WMV for devCCNP-CKG, deveCCNP-PEK and NKI-RS (Fig. 4). These curves indicated rapid increases in WMV from childhood to adolescence consistent with the LBCC findings. To better illustrate the growth curve differences between populations, we depicted site- and sex-specific (adjusted) growth curves of WMV in Fig. 4 (top). WMV is made up of the connections between neurons for cortical communications via neural information flow, and thus, its growth reflects underlying microstructural plasticity during school-age neurodevelopment^84^ (e.g., language performance and training effects during learning^85^). In our analyses, the study-specific variability (e.g., imaging or sample bias) was adjusted by the GAMLSS modelling method. Therefore, the findings we detected are more reproducible and generalizable across devCCNP and NKI-RS samples. Specifically, as shown in Fig. 4, boys had larger WMV than girls, whereas the CKG participants (bottom, right) exhibited relatively smaller WMV than the participants from PEK (bottom, middle) and NKI-RS (bottom, left). Brain growth curves are included in the Supplementary Information (GMV, Figure S1; sGMV, Figure S2; TCV, Figure S3; mean CT, Figure S4; TSA, Figure S5).

**Fig. 5:**
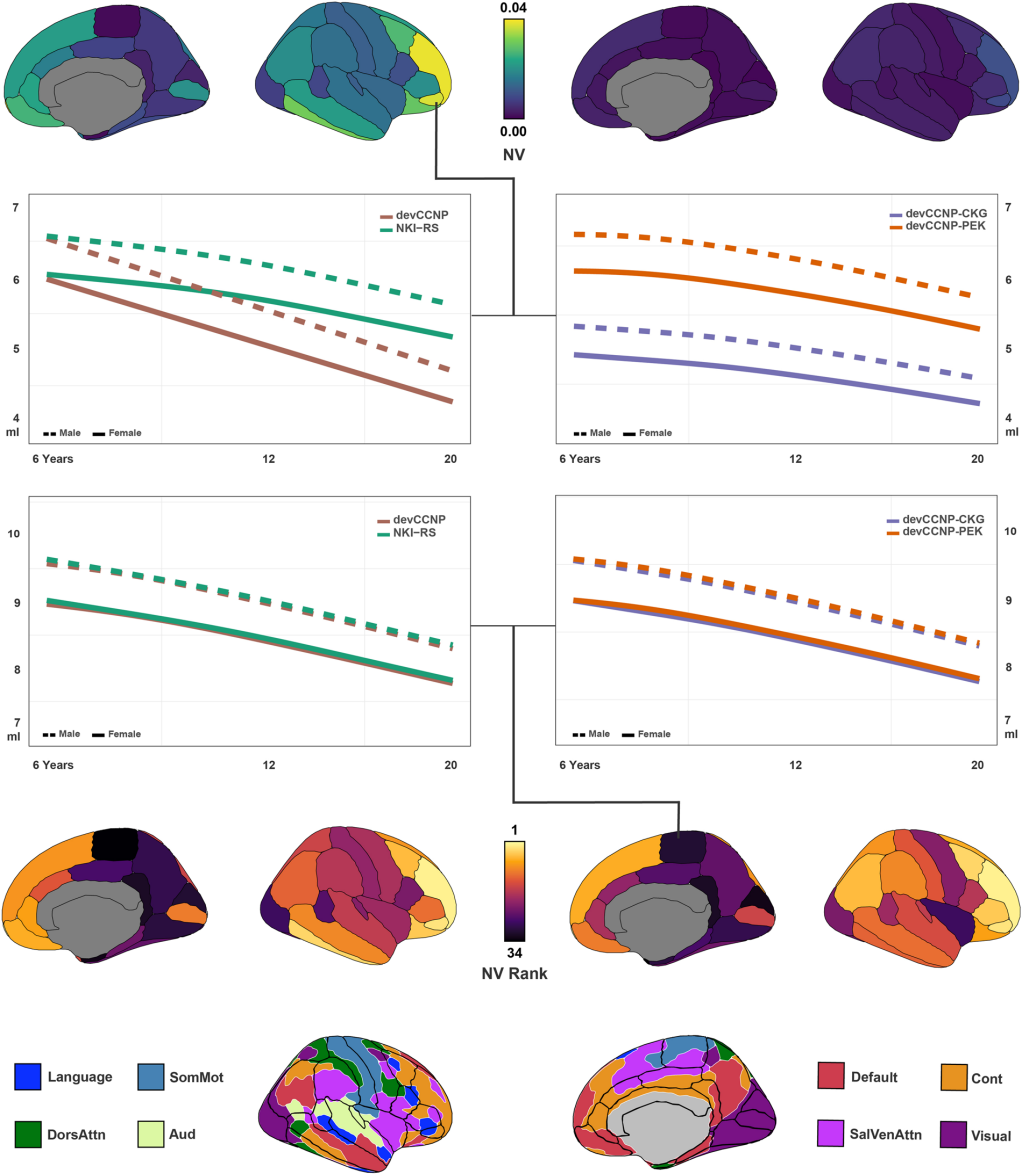
Similarities in brain growth curves between devCCNP and NKI-RS. NV values of the similarity between the United States and China (first row, left) and two samples within devCCNP (first row, right) are presented through 34 gyral-based neuroanatomical regions, referred to as Desikon-Killiany parcellation^83^ (bottom, matched to Kong2022 8 large-scale functional network order^86^). Sex-specific lifespan brain charts of regional volume (unit: *ml*) specific to one high NV (pars orbitalis; second row) and one low NV (paracentral lobule; third row) are depicted. Males are denoted with dashed lines and females are denoted with solid lines. On this basis, the ranks of NV values of these regions are presented (forth row) from highest to lowest. See Supplementary Tables S2, S3 for detailed values. Note that only the NV values and rank of female participants are shown here, as brain charts are modelled sex-specifically^18^. The left hemisphere is plotted here purely for visualization purposes. See Figure S6 for results relating to male participants.

To quantitatively estimate the diversity in brain growth attributable to ethnicity (referring between devCCNP to NKI-RS) and geographics (referring between devCCNP-CKG to devCCNP-PEK), we computed the normalized variance (NV)^16^ of regional volume for each DK-parcel with the following equation

$$NV=2\times \frac{\delta \left({V}_{devCCNP}-{V}_{NKI-RS}\right)}{\mu \left({V}_{devCCNP}+{V}_{NKI-RS}\right)}$$

where *V* is a vector referring to the parcel volume and *δ* referrs to the standard deviation. In other words, NV indicates the degree of curve shape dispersion between two growth curves across ages (we use 0.1 year as the sample age). *δ* is normalized by the mean volume of parcels across two samples, denoted as *μ*. The results are illustrated in Fig. 5 (first row). A small NV indicates that two brain growth curves share similar shapes, and vice versa. The sex-specific lifespan brain charts of regional volume (unit: *ml*) specific to one high NV (Pars Orbitalis; second row) and one low NV (Paracentral Lobule; third row) were depicted to illustrate the differences. As shown in Fig. 5 (bottom), we matched the 34 parcellated regions to the 8 large-scale functional networks^86^ for an intuitive sense of the growth chart differences at the network level. We present the NV rank for comparisons between NKI-RS and devCCNP as well as between devCCNP-CKG and devCCNP-PEK in Fig. 5 (forth row). As done in the LBCC paper^18^, we built normative growth charts of a brain parcel by GAMLSS modelling on the total volume of the parcel as the sum of its two homotopic areas in the two hemispheres. The NV and its rank maps were rendered onto both lateral and medial cortical surfaces of the left hemisphere for visualization purposes. Details of NV are listed in Supplementary Table S2 and S3 according to their ranking orders. Individual differences in growth charts of cortical volumes between devCCNP and NKI-RS are much larger than those between CKG and PEK. Such differences are spatially ranked in a consistent order among populations, indicating more diverse growth curves among individuals in high-order associative (frontoparietal or cognitive control, ventral attention, default mode and language) areas than those in primary areas.

## Usage Notes

Part of this dataset has been successfully used in our previous publications. Two review articles (in Chinese^15^ and English^12^) were published to summarize the devCCNP protocol for experimental design, sample selection, data collection, and preliminary key findings in stages. With the baseline brain imaging data from devCCNP-CKG, we previously reported that children exhibited similar region-specific asymmetry of the dorsal anterior cingulate cortex (dACC) as adults, and further revealed that dACC functional connectivity with the default, frontoparietal and visual networks showed region-specific asymmetry^87^. Head motion data during mock scanning from devCCNP-PEK were used to demonstrate frequency-specific evidence to support motion potentially as a developmental trait in children and adolescents by the development of a neuroinformatic tool DREAM^88^. Social anxiety was positively correlated with the GMV in an area of the orbital-frontal cortex, and its functional connectivity with the amygdala^89^. A standardized protocol for charting brain development in school aged children has been developed to generate the corresponding brain templates and model growth charts, revealing differences in brain morphological growth between Chinese and American populations particularly around puberty^16^. Meanwhile, by manual tracing, we charted the growth curves of the human amygdala across school ages through longitudinal brain imaging^90^. Using rfMRI data, we revealed age-dependent changes in the macroscale organization of the cortex, and the scheduled maturation of functional connectivity gradient shifts, which are critically important for understanding how cognitive and behavioural capabilities are refined across development, marking puberty-related changes^17^.

The baseline imaging data of the CKG Sample has been released as part of the CoRR^77^ and the IPCAS 7 site (10.15387/fcp_indi.corr.ipcas7), which has been listed as one of the existing, ongoing large-scale developmental dataset^91^. As part of an international consortium recently initiated for the generation of human lifespan brain charts^18^, CCNP contributes to the largest worldwide MRI samples (*N* > 120,000) for building normative brain charts for the human lifespan (0–100 years). The full set of devCCNP data is increasingly appreciated by collaborative studies on school-aged children and adolescents. All data obtained freely from the INDI-CoRR-IPCAS7 or CCNC, can only be used for scientific research purposes. The users of this dataset should acknowledge the contributions of the original authors, properly cite the dataset based on the instructions on the Science Data Bank website (10.57760/sciencedb.07478 and 10.57760/sciencedb.07860). We encourage investigators to use this dataset in publication under the requirement of citing this article and contact us for additional data sharing and cooperation.

## Supplementary information

Supplementary Information

## Data Availability

No custom codes or algorithms were used to generate or process the data presented in this manuscript.
