# Supplementary material for: A longitudinal resource for population neuroscience of school-age children and adolescents in China
Source: Sci Data. 2023 Aug 21;10:545. doi: 10.1038/s41597-023-02377-8 (PMC10442366; doi:10.1038/s41597-023-02377-8)
Supplement: Supplementary file 1 — Supplementary Information [file 41597_2023_2377_MOESM1_ESM.pdf]

Supplementary Information: A longitudinal resource for population neuroscience of school-age children and adolescents in China

Xue-Ru Fan (范雪如)[1,2,3,†], Yin-Shan Wang (王银山)[1,3,4,†], Da Chang (常达)[1,3,†], Ning Yang (杨宁)[1,2,3,4], Meng-Jie Rong (荣孟杰)[1,2,3,4], Zhe Zhang (张吉吉)[5], Ye He (何叶)[6], Xiaohui Hou (侯晓晖)[7], Quan Zhou (周荃)[1,2,3], Zhu-Qing Gong (宫竹青)[1,2,3], Li-Zhi Cao (曹立智)[2,4], Hao-Ming Dong (董昊铭)[1,4,8,9], Jing-Jing Nie (聂晶晶)[1,3], Li-Zhen Chen (陈丽珍)[1,3], Qing Zhang (张青)[2,4], Jia-Xin Zhang (张家鑫)[2,4], Lei Zhang (张蕾)[9], Hui-Jie Li (李会杰)[2,4], Min Bao (鲍敏)[2,4], Antao Chen (陈安涛)[10,11], Jing Chen (陈静)[12,13], Xu Chen (陈旭)[11], Jinfeng Ding (丁金丰)[2,4], Xue Dong (董雪)[2,4], Yi Du (杜忆)[2,4], Chen Feng (冯臣)[2,4], Tingyong Feng (冯廷勇)[11], Xiaolan Fu (傅小兰)[2,14], Li-Kun Ge (盖力锟)[2,4], Bao Hong (洪宝)[12,15], Xiaomeng Hu (胡晓檬)[16], Wenjun Huang (黄文君)[12,15], Chao Jiang (蒋超)[17], Li Li (李黎)[12,13], Qi Li (李琦)[17], Su Li (李甦)[2,4], Xun Liu (刘勋)[2,4], Fan Mo (莫凡)[2,14], Jiang Qiu (邱江)[11], Xue-Quan Su (苏学权)[7], Gao-Xia Wei (魏高峡)[2,4], Yiyang Wu (吴伊扬)[2,4], Haishuo Xia (夏海硕)[11], Chao-Gan Yan (严超赣)[2,4], Zhi-Xiong Yan (颜志雄)[7], Xiaohong Yang (杨晓虹)[16], Wenfang Zhang (张文芳)[2,4], Ke Zhao (赵科)[2,14], Liqi Zhu (朱莉琪)[2,4], Lifespan Brain Chart Consortium (LBCC)[*], Chinese Color Nest Consortium (CCNP)[**], and Xi-Nian Zuo (左西年)[1,2,3,4,7,18,*]**

[1]State Key Laboratory of Cognitive Neuroscience and Learning, Beijing Normal University, Beijing, 100875, China.
[2]Department of Psychology, University of Chinese Academy of Sciences, Beijing, 100049, China.
[3]Developmental Population Neuroscience Research Center, International Data Group/McGovern Institute for Brain Research, Beijing Normal University, Beijing, 100875, China.
[4]Key Laboratory of Behavioural Science, Institute of Psychology, Chinese Academy of Sciences, Beijing, 100101, China.
[5]College of Education, Hebei Normal University, Shijiazhuang, 050024, China.
[6]School of Artificial Intelligence, Beijing University of Posts and Telecommunications, Beijing, 100876, China.
[7]Laboratory of Cognitive Neuroscience and Education, School of Education Science, Nanning Normal University, Nanning, 530299, China.
[8]Changping Laboratory, Beijing, 102206, China.
[9]School of Government, Shanghai University of Political Science and Law, Shanghai, 201701, China.
[10]School of Psychology, Research Center for Exercise and Brain Science, Shanghai University of Sport, Shanghai, 200438, China.
[11]Faculty of Psychology, Southwest University, Chongqing, 400715, China.
[12]NYU-ECNU Institute of Brain and Cognitive Science at New York University Shanghai, Shanghai, 200062, China.
[13]Faculty of Arts and Science, New York University Shanghai, Shanghai, 200122, China.
[14]State Key Laboratory of Brain and Cognitive Science, Institute of Psychology, Chinese Academy of Sciences, Beijing, 100101, China.
[15]School of Psychology and Cognitive Science, East China Normal University, Shanghai, 200062, China.
[16]Department of Psychology, Renmin University of China, Beijing, 100872, China.
[17]Beijing Key Laboratory of Learning and Cognition, School of Psychology, Capital Normal University, Beijing, 100048, China.
[18]National Basic Science Data Center, Beijing, 100190, China.
[†]These authors contributed equally to this work as first authors
[*]LBCC is an international consortium and has aggregated 123,984 MRI scans, across more than 100 primary studies, from 101,457 human participants between 115 days post-conception to 100 years of age, and built brain charts to identify previously unreported neurodevelopmental milestones. More information are available at https://github.com/brainchart/lifespan.
[**]CCNP is a long-term effort (2013-2032) to build the lifespan brain-mind development cohort in China, and more consortium information are available at http://deepneuro.bnu.edu.cn/?p=163.

***Corresponding author(s): Xi-Nian Zuo (Website: https://zuoxinian.github.io; Emails: xinian.zuo@bnu.edu.cn, zuoxn@psych.ac.cn, zuoxn@nnnu.edu.cn; Twitter: zuoxinian)

ABSTRACT

During the past decade, cognitive neuroscience has been calling for population diversity to address the challenge of validity and generalizability, ushering in a new era of population neuroscience. The developing Chinese Color Nest Project (devCCNP, 2013–2022), the first ten-year stage of the lifespan CCNP (2013–2032), is a two-stages project focusing on brain-mind development. The project aims to create and share a large-scale, longitudinal and multimodal dataset of typically developing children and adolescents (ages 6.0–17.9 at enrolment) in the Chinese population. The devCCNP houses not only phenotypes measured by demographic, biophysical, psychological and behavioural, cognitive, affective, and ocular-tracking assessments but also neurotypes measured with magnetic resonance imaging (MRI) of brain morphometry, resting-state function, naturalistic viewing function and diffusion structure. This Data Descriptor introduces the first data release of devCCNP including a total of 864 visits from 479 participants. Herein, we provided details of the experimental design, sampling strategies, and technical validation of the devCCNP resource. We demonstrate and discuss the potential of a multicohort longitudinal design to depict normative brain growth curves from the perspective of developmental population neuroscience. The devCCNP resource is shared as part of the "Chinese Data-sharing Warehouse for *In-vivo* Imaging Brain" in the *Chinese Color Nest Project (CCNP) – Lifespan Brain-Mind Development Data Community* (https://ccnp.scidb.cn) at the Science Data Bank.

List of Figures

S1 Site/sex-specific brain charts of grey matter volume (GMV) . . . 3
S2 Site/sex-specific brain charts of subcortical grey matter volume (sGMV) . . . 3
S3 Site/sex-specific brain charts of total cerebrum volume (TCV) . . . 4
S4 Site/sex-specific brain charts of mean cortical thickness (CT) . . . 4
S5 Site/sex-specific brain charts of total surface area (TSA) . . . 5
S6 Similarities of brain growth curves between male participants in devCCNP and NKI-RS . . . 5

List of Tables

S1 Complete protocol . . . 7
S2 NV among female participants in devCCNP and NKI-RS . . . 8
S3 NV among female participants in devCCNP-PEK and devCCNP-CKG . . . 9

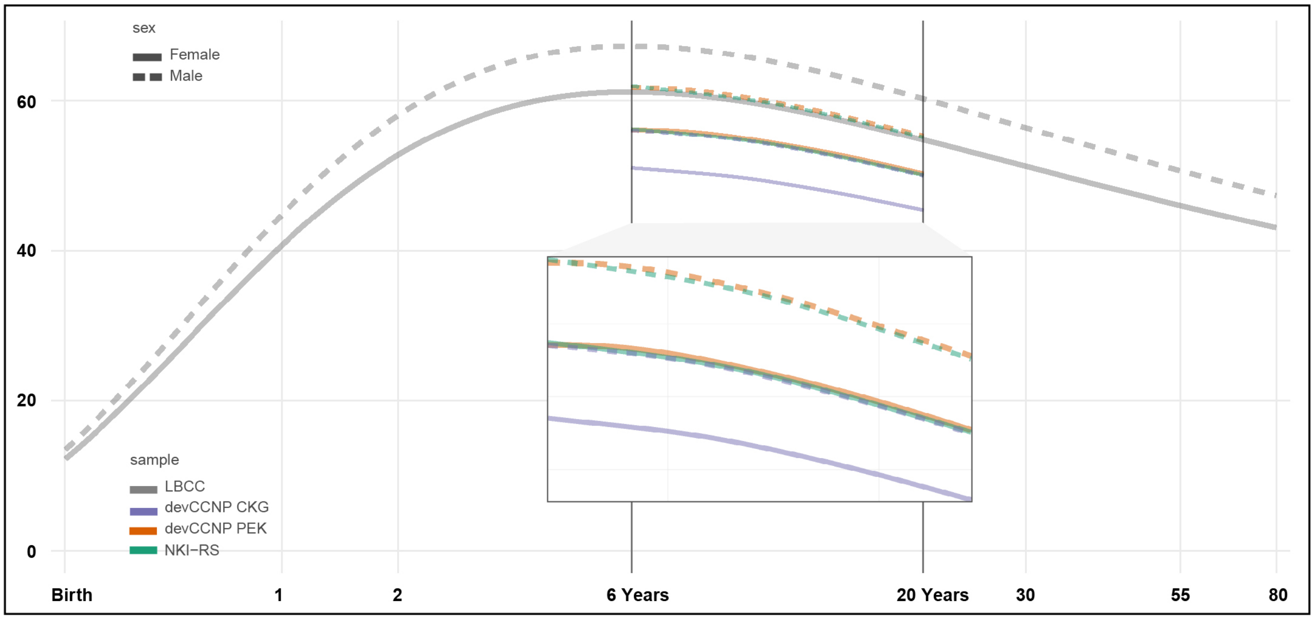

Figure S1. **Site/sex-specific brain charts of grey matter volume (GMV)**

The sex-specific lifespan brain charts of GMV (LBCC, light gray) were adjusted by leveraging the school-aged (6–18 years old) samples for three sites (devCCNP-CKG, purple; devCCNP-PEK, orange; NKI-RS, green), with male (dashed lines) and female (solid lines) respectively. unit: $10ml$ or $10,000mm^3$.

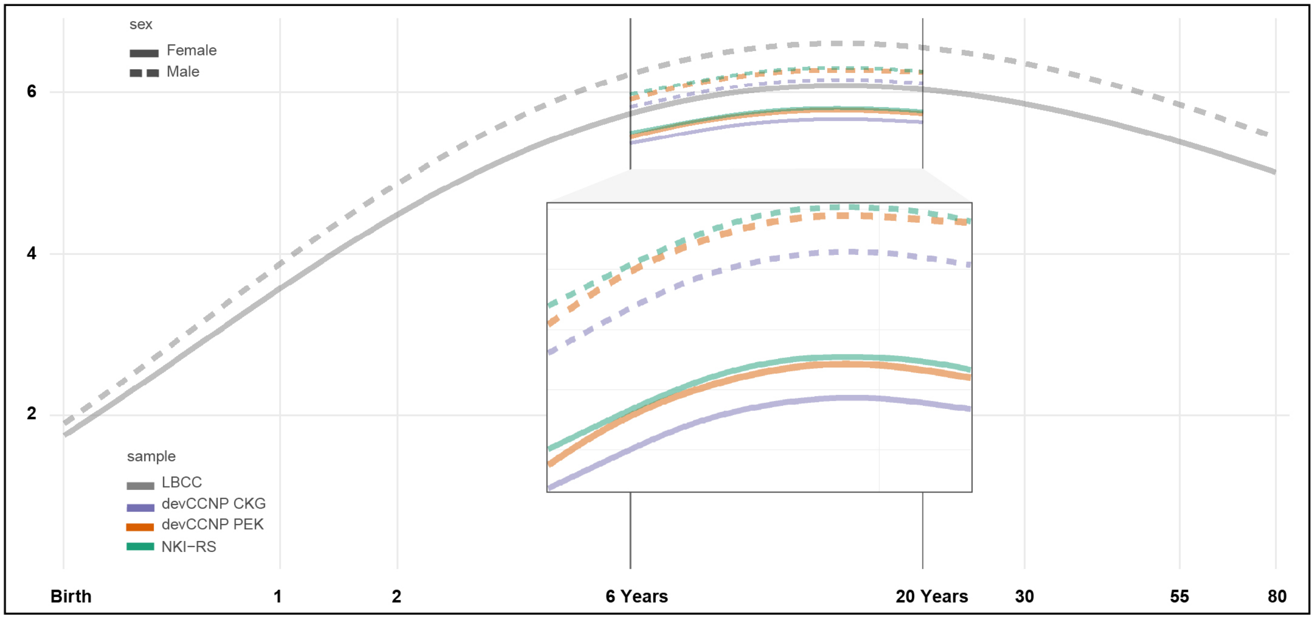

Figure S2. **Site/sex-specific brain charts of subcortical grey matter volume (sGMV)**

The sex-specific lifespan brain charts of sGMV (LBCC, light gray) were adjusted by leveraging the school-aged (6–18 years old) samples for three sites (devCCNP-CKG, purple; devCCNP-PEK, orange; NKI-RS, green), with male (dashed lines) and female (solid lines) respectively. unit: $10ml$ or $10,000mm^3$.

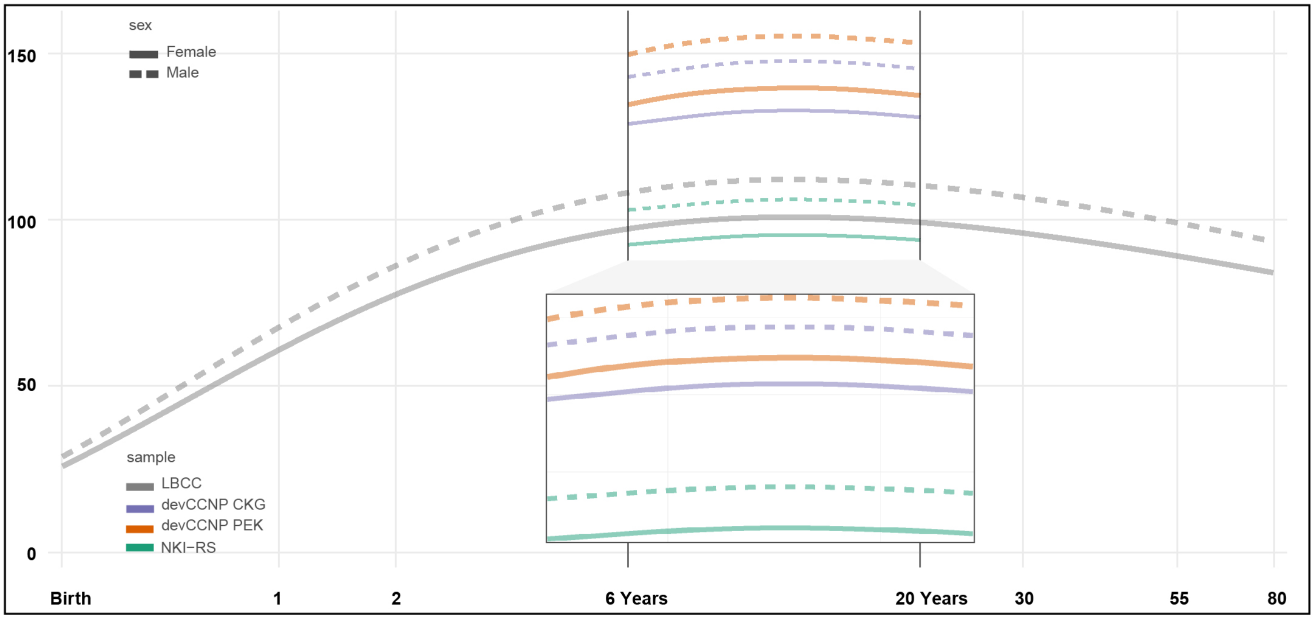

Figure S3. **Site/sex-specific brain charts of total cerebrum volume (TCV)**

The sex-specific lifespan brain charts of TCV (LBCC, light gray) were adjusted by leveraging the school-aged (6–18 years old) samples for three sites (devCCNP-CKG, purple; devCCNP-PEK, orange; NKI-RS, green), with male (dashed lines) and female (solid lines) respectively. unit: $10ml$ or $10,000mm^3$.

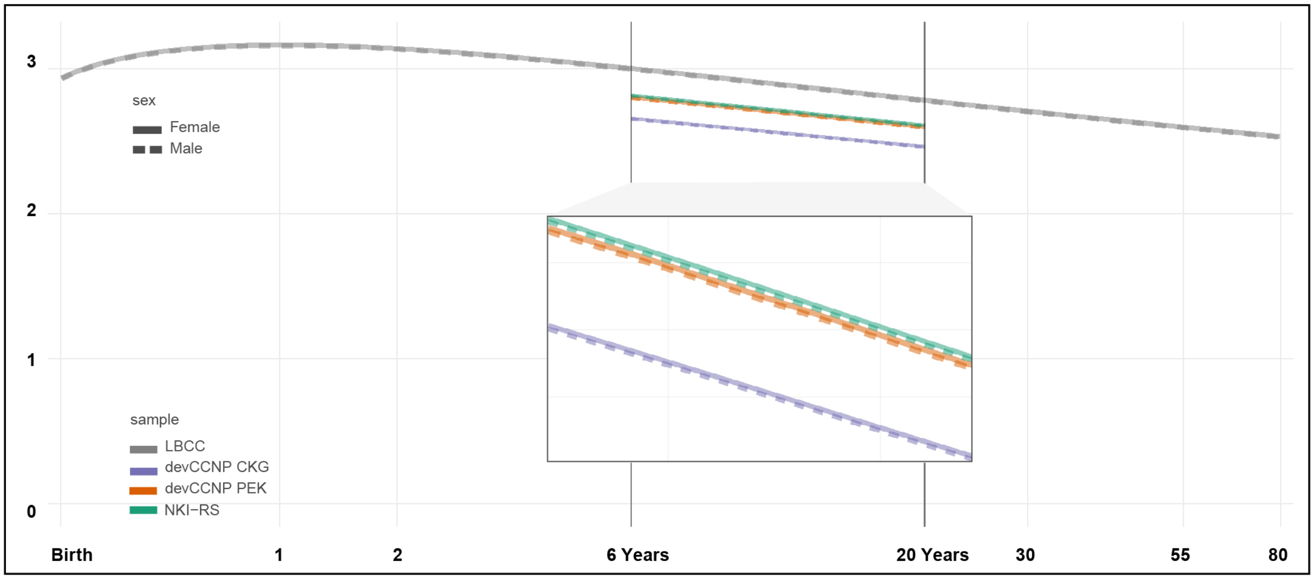

Figure S4. **Site/sex-specific brain charts of mean cortical thickness (CT)**

The sex-specific lifespan brain charts of mean CT (LBCC, light gray) were adjusted by leveraging the school-aged (6–18 years old) samples for three sites (devCCNP-CKG, purple; devCCNP-PEK, orange; NKI-RS, green), with male (dashed lines) and female (solid lines) respectively. unit: *mm*.

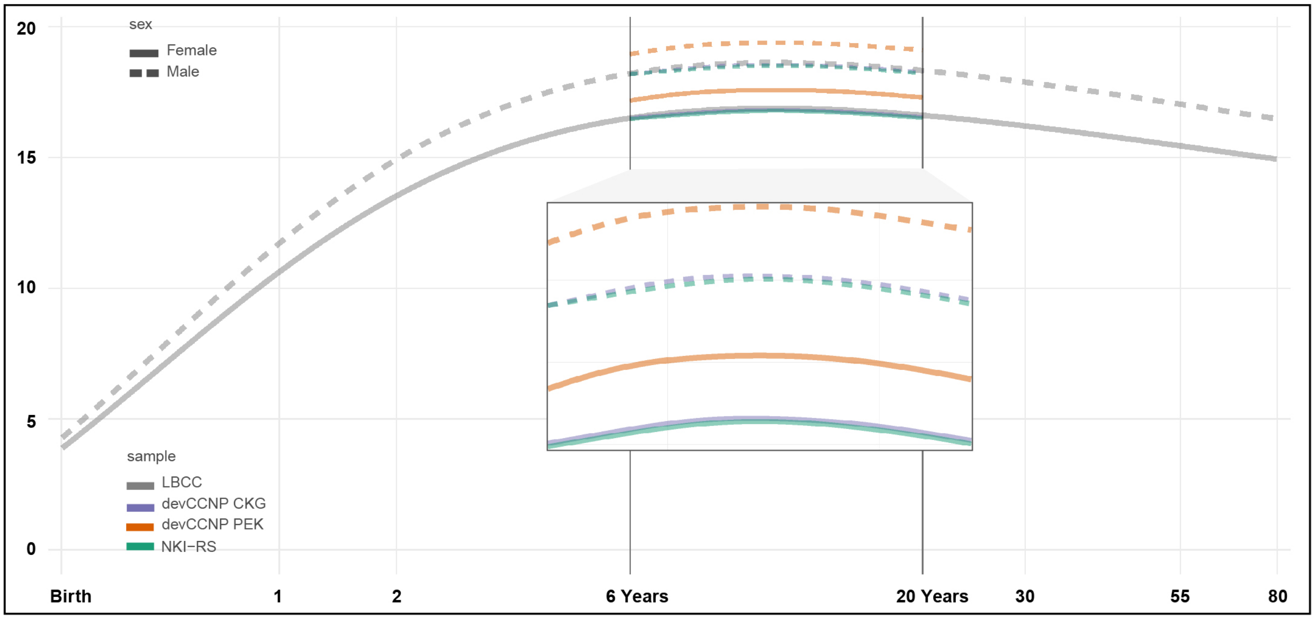

Figure S5. **Site/sex-specific brain charts of total surface area (TSA)**

The sex-specific lifespan brain charts of TSA (LBCC, light gray) were adjusted by leveraging the school-aged (6–18 years old) samples for three sites (devCCNP-CKG, purple; devCCNP-PEK, orange; NKI-RS, green), with male (dashed lines) and female (solid lines) respectively. unit: $10,000mm^2$.

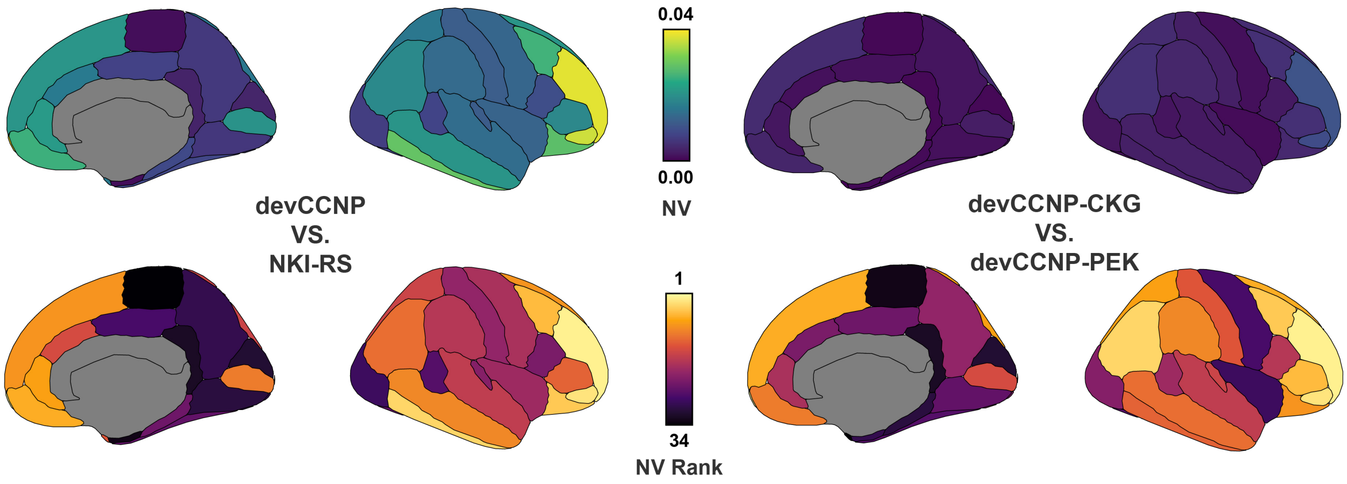

Figure S6. **Similarities of brain growth curves between male participants in devCCNP and NKI-RS**

NV values of the similarity between the United States and China (top, left) and two Samples within devCCNP (top, right) are presented through 34 gyral-based neuroanatomical regions. NV rank of these parcels are presented respectively bottom) from highest (order 1) to lowest (order 34).

	Age of Implementation	CKG Sample	PEK Sample	Duration (minutes)
**Demographics**				
Sex, date of birth, race, birth weight, gestational age at birth	All, Parental	✓	✓	~ 3
Handedness[1,2]	All	✓	✓	
**Participant Characteristic**				
Education level, academic performance	All, Parental	✓	✓	
Music Training History Questionnaire for Children[3,4]	All, Parental		✓	~ 15
Child Behaviour CheckList, CBCL[5,6]	6–18, Parental	✓	✓	
**Family Characteristics**				
Area (urban/rural), number of children	All, Parental	✓	✓	
Education level, careers and industries of parents	All, Parental	✓	✓	~ 3
Subjective social status (Self-designed)	All, Parental		✓	
**Biophysical Measures**				
Height, weight, head circumference, cardiovascular (blood pressure, pulse)	All	✓	✓	~ 3
Visual acuity, pure tone audiometry[7]	6–18		✓	~ 10
**Physical Fitness Measures**				
Grip strength[8], standing broad jump[9], 15-meter shuttle run[10]	6–18		✓	~ 15
Rating of Perceived Exertion, RPE[11]	6–18		✓	~ 1
**Intelligence Quotient Measure**				
Wechsler Intelligence Scale for Children-IV-Chinese Version, WISC-IV[12]	≥ 6	✓	✓	~ 100
Chinese Version of Wechsler Adult Intelligence Scale, WAIS-IV[13]				
**Neuroimaging**				
Mock scan[14]	All		✓	~ 30
Magnetic resonance imaging, MRI	All	✓	✓	~ 50
**Psychological Behaviour Questionnaires**				
Self-reported Pubertal Development Scale (Chinese Version), C-PDS[15,16]	≥ 6		✓	
Pittsburgh Sleep Quality Index, PSQI[17,18]	≥ 6		✓	
Torrance Test of Creative Thinking, TTCT[19,20]	≥ 6	✓		
Williams Creativity Assessment Packet, CAP[20,21]	≥ 6	✓		
Eysenck Personality Questionnaire (Children's Version), EPQ[22,23]	≥ 7	✓	✓	
Eysenck Personality Questionnaire (Audlt's Version), EPQ[22,24]				
Piers-Harris Children's Self-concept Scale, PHCSS[23,25]	6–17	✓	✓	
Social Anxiety Scale for Children, SASC[26,27]	7–16	✓	✓	
Multidimensional Anxiety Scale for Children, MASC[28,29]	8–19	✓	✓	
State-Trait Anxiety Inventory (FormY), STAI-FormY[30,31]	≥ 6	✓	✓	
Chinese Perceived Stress Scale, CPSS[32,33]	> 10	✓	✓	
Positive Affect and Negative Affect Scale, PANAS[34,35]	≥ 6	✓	✓	
Social Value Orientation, SVO[36,37]	≥ 6		✓	
Engagement, Perseverance, Optimism, Connectedness and Happiness Measure (Chinese Version), EPOCH[38,39]	6–18		✓	
				~ 90

Positive Emotion, Engagement, Relationships , Meaning and Accomplishment Profiler, PERMA Profiler[40,41]	≥ 6		✓	
Children's Loneliness Scale, CLS[27,42]	6–12	✓	✓	
Chinese version of Children's Depression Inventory, CDI-C[43,44]	7–17	✓	✓	
Chinese version of the Bar-On Emotional Quotient Inventory: Youth Version, Bar-On EQ-I:YV[45,46]	7–18	✓		
Emotion Regulation Scale, ERS[47,48]	12–18		✓	
Sensation Seeking Scale, SS[49,50]	12–18		✓	
Resilience Scale for Chinese Adolescents, RSCA[51,52]	12–18		✓	
Domain Specific Risk-taking Scale, DOSPERT[53,54]	12–18		✓	
Rosenberg's Self-Esteem Scale, RSES[27,55,56]	12–18		✓	
Social Support Scale, SSS[57,58]	12–18		✓	
Prosocial Tendencies Measure, PTM[59,60]	12–18		✓	
Revised version of Inventory of Parent and Peer Attachment, IPPA-R[61,62]	12–18		✓	
Egna Minnen av Barndoms Uppfostran, EMBU[6,23,63]	12–18		✓	
Adolescent Self-Rating Life Events Checklist, ASLEC[64,65]	13–20	✓	✓	
**Psychological behaviour tasks/tests**				
Attention Network Test[66]	6–18	✓	✓	~ 15
Singleton Stroop Task[67]	≥ 7		✓	~ 15
Task-Switch Paradigm[68]	12–18	✓	✓	~ 5
Digit *N*-back Task[69]	12–18	✓	✓	~ 5
Prisoner's Dilemma[70,71]	7–18		✓	~ 10
Ultimatum Game[72,73]	≥ 7		✓	~ 10
Delay Discounting Task[74]	7–18		✓	~ 5
Risky Decision Task[75,76]	≥ 7		✓	~ 20
Chinese Character Reading Test: Chinese Character Naming Task[77]	6–12	✓	✓	~ 5
Lexical Identification[78,79]	6–18		✓	~ 40
Audiovisual Integration of Words[80,81]	6–18		✓	~ 5
Brief Affect Recognition Test[82,83]	≥ 6		✓	~ 30
Temporal Bisection Paradigm[84,85]	≥ 6		✓	~ 20
Ebbinghaus Illusion[86,87]	≥ 6		✓	~ 15
Binocular Rivalry[88,89]	≥ 6		✓	~ 10
Ocular-tracking Test[90–94]	≥ 6		✓	~ 50
Dichotic Digit Test[95,96]	6–18		✓	~ 5
Competing Sentences[96]	6–18		✓	~ 10
Mandarin Hearing in Noise Test for Children[97]	6–18		✓	~ 20
Verbal Fluency[98,99]	6–18		✓	~ 10

Table S1. Complete protocol

Table S2. **NV among female participants in devCCNP and NKI-RS**

NV Rank	Region	Network	NV
1	Rostral middle frontal gyrus	Default, Language, Cont, SalVenAttn	0.0443
2	Pars orbitalis	Default, Language, Cont	0.0429
3	Inferior temporal gyrus	Default, Language, Cont, SalVenAttn, DorsAttn, Visual	0.0354
4	Lateral orbital frontal cortex	Default, Cont, SalVenAttn	0.0338
5	Caudal middle frontal gyrus	Default, Language, Cont, SalVenAttn, DorsAttn, Visual	0.0310
6	Medial orbital frontal cortex	Default, Cont	0.0299
7	Rostral anterior cingulate cortex	Default, Cont	0.0263
8	Superior frontal gyrus	Default, Language, Cont, SalVenAttn, DorsAttn, SomMot	0.0251
9	Middle temporal gyrus	Default, Language, Cont, SalVenAttn, DorsAttn, Aud, Visual	0.0249
10	Pericalcarine cortex	Visual	0.0230
11	Pars triangularis	Default, Language, Cont, SalVenAttn	0.0224
12	Inferior parietal cortex	Default, Cont, SalVenAttn, DorsAttn, Aud, Visual	0.0223
13	Caudal anterior-cingulate cortex	Cont, SalVenAttn	0.0197
14	Temporal pole	Default	0.0188
15	Superior parietal cortex	Cont, DorsAttn, SomMot, Visual	0.0181
16	Superior temporal gyrus	Default, Language, SalVenAttn, Aud	0.0171
17	Supramarginal gyrus	Cont, SalVenAttn, DorsAttn, SomMot, Aud	0.0167
18	Precentral gyrus	Language, SalVenAttn, DorsAttn, SomMot, Visual	0.0158
19	Postcentral gyrus	SalVenAttn, DorsAttn, SomMot	0.0140
20	Transverse temporal cortex	Aud	0.0137
21	Pars opercularis	Language, Cont, SalVenAttn, DorsAttn	0.0114
22	Parahippocampal gyrus	Default, Cont, DorsAttn, Visual	0.0104
23	Fusiform gyrus	Default, Language, SalVenAttn, DorsAttn, Visual	0.0101
24	Banks superior temporal sulcus	Default, Language, SalVenAttn, Aud	0.0096
25	Posterior-cingulate cortex	Default, Cont, SalVenAttn, SomMot	0.0089
26	Lateral occipital cortex	Visual	0.0084
27	Precuneus cortex	Default, Cont, SalVenAttn, DorsAttn, SomMot, Visual	0.0072
28	Lingual gyrus	Cont, Visual	0.0071
29	Cuneus cortex	Visual	0.0042
30	Isthmus–cingulate cortex	Default, Cont	0.0039
31	Entorhinal cortex	Default, DorsAttn	0.0011
32	Paracentral lobule	SalVenAttn, SomMot, Visual	0.0009

[1] As explained in the manual delineation procedure of Desikon-Killiany parcellation, the region frontal pole was not actually designed as a measure of the frontal pole itself. Other frontal lobe regions were first designated and the remaining portion was called the frontal pole, which was also proven to be unreliable. The region corpus callosum was introduced to better define the regions around it. Therefore, the NV of these two regions is not shown. Note this table only refer to female participants. The text color corresponding to the large-scale network illustrated in Figure 5.)

Table S3. **NV among female participants in devCCNP-PEK and devCCNP-CKG**

NV Rank	Region	Network	NV
1	Rostral middle frontal gyrus	Default, Language, Cont, SalVenAttn	0.0114
2	Pars orbitalis	Default, Language, Cont	0.0101
3	Caudal middle frontal gyrus	Default, Language, Cont, SalVenAttn, DorsAttn, Visual	0.0056
4	Pars triangularis	Default, Language, Cont, SalVenAttn	0.0055
5	Inferior parietal cortex	Default, Cont, SalVenAttn, DorsAttn, Aud, Visual	0.0054
6	Superior frontal gyrus	Default, Language, Cont, SalVenAttn, DorsAttn, SomMot	0.0051
7	Lateral orbital frontal cortex	Default, Cont, SalVenAttn	0.0045
8	Superior parietal cortex	Cont, DorsAttn, SomMot, Visual	0.0045
9	Supramarginal gyrus	Cont, SalVenAttn, DorsAttn, SomMot, Aud	0.0039
10	Medial orbital frontal cortex	Default, Cont	0.0039
11	Middle temporal gyrus	Default, Language, Cont, SalVenAttn, DorsAttn, Aud, Visual	0.0036
12	Inferior temporal gyrus	Default, Language, Cont, SalVenAttn, DorsAttn, Visual	0.0036
13	Postcentral gyrus	SalVenAttn, DorsAttn, SomMot	0.0029
14	Superior temporal gyrus	Default, Language, SalVenAttn, Aud	0.0026
15	Pericalcarine cortex	Visual	0.0026
16	Transverse temporal cortex	Aud	0.0026
17	Pars opercularis	Language, Cont, SalVenAttn, DorsAttn	0.0025
18	Rostral anterior cingulate cortex	Default, Cont	0.0024
19	Banks superior temporal sulcus	Default, Language, SalVenAttn, Aud	0.0020
20	Caudal anterior-cingulate cortex	Cont, SalVenAttn	0.0019
21	Precentral gyrus	Language, SalVenAttn, DorsAttn, SomMot, Visual	0.0018
22	Lateral occipital cortex	Visual	0.0017
23	Fusiform gyrus	Default, Language, SalVenAttn, DorsAttn, Visual	0.0017
24	Precuneus cortex	Default, Cont, SalVenAttn, DorsAttn, SomMot, Visual	0.0015
25	Posterior-cingulate cortex	Default, Cont, SalVenAttn, SomMot	0.0014
26	Parahippocampal gyrus	Default, Cont, DorsAttn, Visual	0.0013
27	Lingual gyrus	Cont, Visual	0.0011
28	Entorhinal cortex	Default, DorsAttn	0.0011
29	Paracentral lobule	SalVenAttn, SomMot, Visual	0.0008
30	Isthmus–cingulate cortex	Default, Cont	0.0006
31	Cuneus cortex	Visual	0.0005
32	Temporal pole	Default	0.0003

References

1. Annett, M. A classification of hand preference by association analysis. *Br. J. Psychol.* **61**, 303–321 (1970).
2. Oldfield, R. C. The assessment and analysis of handedness: the Edinburgh inventory. *Neuropsychologia* **9**, 97–113 (1971).
3. Zhang, L., Fu, X., Luo, D., Xing, L. & Du, Y. Musical Experience Offsets Age-Related Decline in Understanding Speech-in-Noise: Type of Training Does Not Matter, Working Memory Is the Key. *Ear Hear.* **42**, 258–270 (2021).
4. Coffey, E. B. J., Herholz, S. C., Scala, S. & Zatorre, R. J. Montreal Music History Questionnaire: a tool for the assessment of music-related experience in music cognition research. In *The Neurosciences and Music IV: Learning and Memory, Conference* (Edinburgh, UK, 2011).
5. Su, L., Li, X., Luo, X., Wan, G. & Yang, Z. The Newly Revised Norms of Child Behavior Checklist in Hunan Province (in Chinese). *Chin. Mental Heal. J.* **12**, 67–69 (1998).
6. Yu, X. *et al.* Internalizing Behavior Problems Among the Left-Behind Children of the Hui Nationality in Rural China: A Cross-Sectional Study. *Psychol. Res. Behav. Manag.* **15**, 887–902 (2022).
7. Working Group on Manual Pure-Tone Threshold Audiometry. Guidelines for manual pure-tone threshold audiometry. *Am. Speech-Language-Hearing Assoc.* (2005).
8. Wind, A. E., Takken, T., Helders, P. J. & Engelbert, R. H. Is grip strength a predictor for total muscle strength in healthy children, adolescents, and young adults? *Eur. J. Pediatr.* **169**, 281–287 (2010).
9. Korff, T., Horne, S. L., Cullen, S. J. & Blazevich, A. J. Development of lower limb stiffness and its contribution to maximum vertical jumping power during adolescence. *J. Exp. Biol.* **212**, 3737–3742 (2009).
10. Jackson, A. S., Sui, X., Hebert, J. R., Church, T. S. & Blair, S. N. Role of Lifestyle and Aging on the Longitudinal Change in Cardiorespiratory Fitness. *Arch. Intern. Medicine* **169**, 1781–1787 (2009).
11. Andersen, L. B. *et al.* Physical activity and clustered cardiovascular risk in children: a cross-sectional study (The European Youth Heart Study). *Lancet* **368**, 299–304 (2006).
12. Zhang, H. C. The revision of WISC-IV Chinese version (in Chinese). *Psychol. Sci.* **32**, 1177–1179 (2009).
13. Cui, J. F. *et al.* Norm development of the Chinese edition of Wechsler Adult Intelligence Scale-Fourth Edition (in Chinese). *Chin. Mental Heal. J.* **31**, 635–641 (2017).
14. Suzuki, A., Yamaguchi, R., Kim, L., Kawahara, T. & Ishii-Takahashi, A. Effectiveness of mock scanners and preparation programs for successful magnetic resonance imaging: a systematic review and meta-analysis. *Pediatr. Radiol.* **53**, 142–158 (2023).
15. Zhu, L. & Chen, P. Verification of the Self-reported Pubertal Development Scale (Chinese Version) (in Chinese). *Chin. J. Sports Medicine* **21**, 512–516 (2012).
16. Petersen, A. C., Crockett, L., Richards, M. & Boxer, A. A self-report measure of pubertal status: Reliability, validity, and initial norms. *J. Youth Adolesc.* **17**, 117–133 (1988).
17. Lu, T., Yan, L., Ping, X., Zhang, G. & Wu, D. Analysis on reliability and validity of the Pittsburgh sleep quality index (in Chinese). *Chongqing Medicine* **43**, 260–263 (2014).
18. Buysse, D. J., Reynolds, r., C. F., Monk, T. H., Berman, S. R. & Kupfer, D. J. The Pittsburgh Sleep Quality Index: a new instrument for psychiatric practice and research. *Psychiatry Resarch* **28**, 193–213 (1989).
19. Ye, R., Hong, D. & Torrance, P. E. Cross Cultural Comparison of Creative Thinking between Chinese and American Students Using Torrance Test (in Chinese). *Chin. J. Appl. Psychol.* **3**, 22–29 (1988).
20. Cooper, E. A Critique of 6 Measures for Assessing Creativity. *J. Creat. Behav.* **25**, 194–204 (1991).
21. Liu, X. L., Liu, L., Qiu, Y. X., Jin, Y. & Zhou, J. Reliability and Validity of Williams Creativity Assessment Packet (in Chinese). *J. Sch. Stud.* **13**, 51–58 (2016).
22. Gong, Y. Eysenck Personality Questionnaire Revised in China (in Chinese). *J. Psychol. Sci.* **4**, 13–20,67 (1984).
23. Yu, X., Wang, L., Liu, M., Li, Q. & Dai, X. Externalizing Behavior Problems Among Hui Ethnicity Left-Behind Children in Rural China: A Cross-Sectional Study. *Psychiatry Investig* **19**, 289–301 (2022).
24. Su, Q. & Liu, G. F. A cross-temporal meta-analysis review of the personality of Chinese military personnel, 1991-2017. *Pers. Mental Heal.* **15**, 124–135 (2021).
25. Su, L., Luo, X., Zhang, J., Xie, G. & Liu, Y. Norms of the Piers-Harris Children's Self-concept Scale of Chinese Urban Children (in Chinese). *Chin. Mental Heal. J.* **16**, 31–34 (2002).

26. Li, F., Su, L. Y. & Jin, Y. Norm of the screen for child social anxiety related emotional disorders in Chinese urban children (in Chinese). *Chin. J. Child Heal. Care* **14**, 335–337 (2006).

27. Chen, C. & Hu, L. Self-esteem mediated relations between loneliness and social anxiety in Chinese adolescents with left-behind experience. *Front. Psychol.* **13**, 1014794 (2022).

28. Zou, T., Yao, S. Q., Zhu, X. Z., Abela, J. R. Z. & Auerbach, R. P. Reliability and validity of the Chinese version of the multidimensional anxiety scale for Chinese (in Chinese). *Chin. J. Clin. Psychol.* **15**, 452–455 (2007).

29. Thaler, N. S., Kazemi, E. & Wood, J. J. Measuring anxiety in youth with learning disabilities: reliability and validity of the Multidimensional Anxiety Scale for Children (MASC). *Child Psychiatry & Hum. Dev.* **41**, 501–514, 10.1007/s10578-010-0182-5 (2010).

30. Li, W. & Qian, M. Revision of the State-Trait anxiety inventory with sample of Chinese college students (in Chinese). *Acta Sci. Nat. Univ. Pekinensis* **31**, 108–112 (1995).

31. Wang, K. *et al.* Cross-cultural validation of the Depression Anxiety Stress Scale-21 in China. *Psychol. Assess.* **28**, e88–e100 (2016).

32. Cohen, S., Kamarck, T. & Mermelstein, R. A Global Measure of Perceived Stress. *J. Heal. Soc. Behav.* **24**, 385–396 (1983).

33. Lu, W. *et al.* Chinese version of the Perceived Stress Scale-10: A psychometric study in Chinese university students (in Chinese). *PLoS One* **12**, e0189543 (2017).

34. Huang, L., Yang, T. & Li, Z. Applicability of the Positive and Negative Affect Scale in Chinese (in Chinese). *Chin. Mental Heal. J.* **17**, 54–56 (2003).

35. Wolniewicz, C. A., Tiamiyu, M. F., Weeks, J. W. & Elhai, J. D. Problematic smartphone use and relations with negative affect, fear of missing out, and fear of negative and positive evaluation. *Psychiatry Res.* **262**, 618–623 (2018).

36. Murphy, R. O., Ackermann, K. A. & Handgraaf, M. Measuring Social Value Orientation. *Judgm. Decis. Mak.* **6**, 771–781 (2011).

37. Cui, M.-S., Zhang, X.-K. & Ding, X.-P., Xiang-Ling. Deng. Development and Psychometric Analysis of the SVO Slider Measure in Adolescence (in Chinese). *Chin. J. Clin. Psychol.* **26**, 272–276 (2018).

38. Kern, M. L., Zeng, G., Hou, H. & Peng, K. The Chinese Version of the EPOCH Measure of Adolescent Well-Being: Testing Cross-Cultural Measurement Invariance. *J. Psychoeduc. Assess.* **37**, 757–769 (2019).

39. Kern, M. L., Benson, L., Steinberg, E. A. & Steinberg, L. The EPOCH Measure of Adolescent Well-Being. *Psychol. Assess.* **28**, 586–597 (2016).

40. Butler, J. & Kern, M. L. The PERMA-Profiler: A brief multidimensional measure of flourishing. *Int. J. Wellbeing* **6**, 1–48 (2016).

41. Kern, M. L., Waters, L. E., Adler, A. & White, M. A. A multidimensional approach to measuring well-being in students: Application of the PERMA framework. *J. Posit. Psychol.* **10**, 262–271 (2015).

42. Gao, J. J. & Chen, Y. W. Applicability of the Children's Loneliness Scale in 1-2 grade pupils (in Chinese). *Chin. Mental Heal. J.* **25**, 361–364 (2011).

43. Wu, W. F., Lu, Y. B., Tan, F. R. & Yao, S. Q. Reliability and validity of the Chinese version of Children's Depression Inventory (in Chinese). *Chin. Mental Heal. J.* **24**, 775–779 (2010).

44. Duan, L. *et al.* An investigation of mental health status of children and adolescents in china during the outbreak of COVID-19. *J. Affect. Disord.* **275**, 112–118 (2020).

45. Zhao, Q., Jia, W., Ying, B., Psychology, S. O. & University, S. Reliability and Validity of Chinese Version of Bar-On Emotional Quotient Inventory: Youth Version (in Chinese). *China J. Heal. Psychol.* **21**, 1511–1515 (2013).

46. Conte, J. M. A review and critique of emotional intelligence measures. *J. Organ. Behav.* **26**, 433–440 (2005).

47. Gross, J. J. & John, O. P. Individual differences in two emotion regulation processes: Implications for affect, relationships, and well-being. *J. Pers. Soc. Psychol.* **85**, 348–362 (2003).

48. Wang, L., Liu, H., Li, Z. & Du, W. Reliability and validity of the emotion regulation scale (in Chinese). *China J. Heal. Psychol.* **16**, 846–848 (2007).

49. Chen, X. G. *et al.* Brief Sensation Seeking Scale for Chinese - Cultural adaptation and psychometric assessment. *Pers. Individ. Differ.* **54**, 604–609 (2013).

50. Stautz, K. & Cooper, A. Impulsivity-related personality traits and adolescent alcohol use: a meta-analytic review. *Clin. Psychol. Rev.* **33**, 574–592 (2013).

51. Hu, Y.-Q. & Gan, Y.-Q. Development and Psychometric Validity of the Resilience Scale for Chinese Adolescents (in Chinese). *Acta Psychol. Sinica* **40**, 902–912 (2008).

52. Wen, Y. *et al.* Mental resilience tested with the Resilience Scale for Chinese Adolescents (RSCA) in Chinese children: A meta-analysis (in Chinese). *Chin. Mental Heal. J.* **11**, 826–832 (2015).

53. Shang, L. & Zhang, L. Construction and Application of both Adolescent Multi-domain Risk Behavior Questionnaire and Risk Perception Questionnaire (in Chinese). *Chin. J. Epidemiol.* **32**, 571–575 (2011).

54. Hu, X. X. & Xie, X. F. Validation of the Domain-Specific Risk-Taking Scale in Chinese college students. *Judgm. Decis. Mak.* **7**, 181–188 (2012).

55. Yang, Y. & Wang, D. Retest of the Bidimensional Model of Rosenberg Self-Esteem Scale (in Chinese). *Chin. Mental Heal. J.* **21**, 603–605,609 (2007).

56. Tian, L. Shortcoming and merits of Chinese version of Rosenberg (1965) Self-Esteem Scale (in Chinese). *Psychol. Explor.* **26**, 88–91 (2006).

57. Luo, J. & Dai, X.-y. A Generalizability Analysis of the Social Support Scale for University Students (in Chinese). *Chin. J. Clin. Psychol.* **19**, 181–183 (2011).

58. Kong, F. & You, X. Q. Loneliness and Self-Esteem as Mediators Between Social Support and Life Satisfaction in Late Adolescence. *Soc. Indic. Res.* **110**, 271–279 (2013).

59. Kou, Y., Hong, H. F., Tan, C. & Li, L. Revisioning Prosocial Tendencies Measure for Adolescent (in Chinese). *Psychol. Dev. Educ.* **23**, 112–117 (2007).

60. Carlo, G. & Randall, B. A. The development of a measure of prosocial behaviors for late adolescents. *J. Youth Adolesc.* **31**, 31–44 (2002).

61. Zhang, Y. L., Zhang, Y. L., Zhang, Y. X., Wang, J. L. & Huang, C. Y. Reliability and validity of Chinese version of Revised Inventory of Parent and Peer Attachment in junior students (in Chinese). *Chin. Mental Heal. J.* **25**, 66–70 (2011).

62. Gullone, E. & Robinson, K. The inventory of parent and peer attachment - Revised (IPPA-R) for children: A psychometric investigation. *Clin. Psychol. & Psychother.* **12**, 67–79 (2005).

63. Dongmei, Y., Li, M., Jin, K. & Ding, B. Preliminary revision of EMBU and its application in neurotic patients (in Chinese). *Chin. Mental Heal. J.* **3**, 97–101,143 (1993).

64. Xin, X. H. & Yao, S. Q. Validity and reliability of the Adolescent Self-rating Life Events Checklist in middle school students (in Chinese). *Chin. Mental Heal. J.* **29**, 355–360 (2015).

65. Zhao, F. *et al.* The association between life events and internet addiction among Chinese vocational school students: The mediating role of depression. *Comput. Hum. Behav.* **70**, 30–38 (2017).

66. Fan, J., McCandliss, B. D., Sommer, T., Raz, A. & Posner, M. I. Testing the efficiency and independence of attentional networks. *J. Cogn. Neurosci.* **14**, 340–347 (2002).

67. Fu, D. & Liu, X. Interaction between Attentional Capture and Executive Control in Conflict Processing (in Chinese). Preprint at https://psyarxiv.com/8chkn (2020).

68. Schuch, S. & Koch, I. The role of response selection for inhibition of task sets in task shifting. *J. Exp. Psychol. Hum. Percept. Perform.* **29**, 92–105 (2003).

69. Zhu, D. F. *et al.* fMRI revealed neural substrate for reversible working memory dysfunction in subclinical hypothyroidism. *Brain* **129**, 2923–2930 (2006).

70. Molina, J. A. *et al.* Gender Differences in Cooperation: Experimental Evidence on High School Students. *PLoS One* **8**, e83700 (2013).

71. Cardenas, J. C., Dreber, A., von Essen, E. & Ranehill, E. Gender and cooperation in children: experiments in Colombia and Sweden. *PLoS One* **9**, e90923 (2014).

72. Takagishi, H. *et al.* The Role of Cognitive and Emotional Perspective Taking in Economic Decision Making in the Ultimatum Game. *PLoS One* **9**, e108462 (2014).

73. Li, Y. Y. *et al.* The development of inequity aversion in Chinese children. *Cogn. Dev.* **61** (2022).

74. Achterberg, M., Peper, J. S., van Duijvenvoorde, A. C., Mandl, R. C. & Crone, E. A. Frontostriatal White Matter Integrity Predicts Development of Delay of Gratification: A Longitudinal Study. *J. Neurosci.* **36**, 1954–1961 (2016).

75. Zheng, Y. *et al.* Deficits in voluntary pursuit and inhibition of risk taking in sensation seeking. *Hum. Brain Mapp.* **38**, 6019–6028 (2017).

76. Wang, L. X. *et al.* Enhanced neural responses in specific phases of reward processing in individuals with Internet gaming disorder. *J. Behav. Addict.* **10**, 99–111 (2021).

77. Xue, J., Shu, H., Li, H., Li, W. & Tian, X. The stability of literacy-related cognitive contributions to Chinese character naming and reading fluency. *J. Psycholinguist. Res.* **42**, 433–450 (2013).

78. Plaut, D. C. & Booth, J. R. Individual and developmental differences in semantic priming: empirical and computational support for a single-mechanism account of lexical processing. *Psychol. Rev.* **107**, 786–823 (2000).

79. Mirman, D., Landrigan, J. F. & Britt, A. E. Taxonomic and Thematic Semantic Systems. *Psychol. Bull.* **143**, 499–520 (2017).

80. Xu, W., Kolozsvari, O. B., Oostenveld, R., Leppanen, P. H. T. & Hamalainen, J. A. Audiovisual Processing of Chinese Characters Elicits Suppression and Congruency Effects in MEG. *Front. Hum. Neurosci.* **13**, 18 (2019).

81. Jost, L. B., Eberhard-Moscicka, A. K., Frisch, C., Dellwo, V. & Maurer, U. Integration of Spoken and Written Words in Beginning Readers: A Topographic ERP Study. *Brain Topogr.* **27**, 786–800 (2014).

82. Zhao, K., Zhao, J., Zhang, M., Cui, Q. & Fu, X. L. Neural Responses to Rapid Facial Expressions of Fear and Surprise. *Front. Psychol.* **8**, 761 (2017).

83. Mancini, G., Agnoli, S., Baldaro, B., Bitti, P. E. R. & Surcinelli, P. Facial Expressions of Emotions: Recognition Accuracy and Affective Reactions During Late Childhood. *J. Psychol.* **147**, 599–617 (2013).

84. Droit-Volet, S. & Rattat, A. C. A further analysis of time bisection behavior in children with and without reference memory: The similarity and the partition task. *Acta Psychol.* **125**, 240–256 (2007).

85. Droit-Volet, S. & Wearden, J. Speeding up an internal clock in children? Effects of visual flicker on subjective duration. *Q. J. Exp. Psychol. Sect. B-Comparative Physiol. Psychol.* **55**, 193–211 (2002).

86. Weintraub, D. J. Ebbinghaus Illusion - Context, Contour, and Age Influence the Judged Size of a Circle Amidst Circles. *J. Exp. Psychol. Percept. Perform.* **5**, 353–364 (1979).

87. Hadad, B. S. Developmental trends in susceptibility to perceptual illusions: Not all illusions are created equal. *Atten. Percept. & Psychophys.* **80**, 1619–1628 (2018).

88. Fagard, J., Monzalvo-Lopez, K. & Mamassian, P. Relationship Between Eye Preference and Binocular Rivalry, and Between Eye-Hand Preference and Reading Ability in Children. *Dev. Psychobiol.* **50**, 789–798 (2008).

89. Lunghi, C., Morrone, M. C., Secci, J. & Caputo, R. Binocular Rivalry Measured 2 Hours After Occlusion Therapy Predicts the Recovery Rate of the Amblyopic Eye in Anisometropic Children. *Investig. Ophthalmol. & Vis. Sci.* **57**, 1537–1546 (2016).

90. Liston, D. B. & Stone, L. S. Oculometric assessment of dynamic visual processing. *J. Vis.* **14**, 1–17 (2014).

91. Stone, L. S., Tyson, T. L., Cravalho, P. F., Feick, N. H. & Flynn-Evans, E. E. Distinct pattern of oculomotor impairment associated with acute sleep loss and circadian misalignment. *J. Physiol.* **597**, 4643–4660 (2019).

92. Tyson, T. L., Feick, N. H., Cravalho, P. F., Flynn-Evans, E. E. & Stone, L. S. Dose-dependent sensorimotor impairment in human ocular tracking after acute low-dose alcohol administration. *J. Physiol.* **599**, 1225–1242 (2021).

93. Chen, R. R., Stone, L. S. & Li, L. Visuomotor predictors of batting performance in baseball players. *J. Vis.* **21**, 1–16 (2021).

94. Chen, J. *et al.* Impaired Ocular Tracking and Cortical Atrophy in Idiopathic Rapid Eye Movement Sleep Behavior Disorder. *Mov. Disord.* **37**, 972–982 (2022).

95. Cameron, S., Glyde, H., Dillon, H., Whitfield, J. & Seymour, J. The Dichotic Digits difference Test (DDdT): Development, Normative Data, and Test-Retest Reliability Studies Part 1. *J. Am. Acad. Audiol.* **27**, 458–469 (2016).

96. Liu, Q. *et al.* A Comparative Study on Auditory Processing Abilities between Children with and without Learning Difficulties (in Chinese). *J. Audiol. Speech Pathol.* **25**, 14–18 (2017).

97. Ning, Z. *et al.* The Establishment of Age-Specific Correction Factors of Mandarin Hearing in Noise Test for Children (in Chinese). *J. Audiol. Speech Pathol.* **20**, 97–101 (2012).

98. Riva, D., Nichelli, F. & Devoti, M. Developmental aspects of verbal fluency and confrontation naming in children. *Brain Lang.* **71**, 267–284 (2000).

99. Cohen, M. J., Morgan, A. M., Vaughn, M., Riccio, C. A. & Hall, J. Verbal fluency in children: Developmental issues and differential validity in distinguishing children with attention-deficit hyperactivity disorder and two subtypes of dyslexia. *Arch. Clin. Neuropsychol.* **14**, 433–443 (1999).
